# Simulation-based assessment of a Bayesian M-spline survival model with flexible baseline hazard and time-dependent effects

**DOI:** 10.1186/s12874-026-02783-7

**Published:** 2026-02-12

**Authors:** Iain R. Timmins, Fatemeh Torabi, Christopher H. Jackson, Paul C. Lambert, Michael J. Sweeting

**Affiliations:** 1 Statistical Innovation, Oncology R&D, AstraZeneca, Cambridge, UK; 2 Department of Health Services Research and Policy, London School of Hygiene & Tropical Medicine, London, UK; 3 Professional and Continuing Education, University of Cambridge, Cambridge, UK; 4 Dementias Platform UK, Swansea University, Swansea, Wales UK; 5 MRC Biostatistics Unit, University of Cambridge, Cambridge, UK; 6 Cancer Registry of Norway, Norwegian Institute of Public Health, Oslo, Norway; 7 Department of Medical Epidemiology and Biostatistics, Karolinska Institutet, Stockholm, Sweden; 8 Department of Population Health Sciences, University of Leicester, Leicester, UK

**Keywords:** Bayesian, Survival analysis, Clinical trials, HTA, MCMC, Parametric models, Spline estimator, Hazard function, Statistical software

## Abstract

**Background:**

There is increasing interest in flexible Bayesian models for the analysis of time-to-event data, especially with their use in medical applications such as Health Technology Assessment (HTA). While these Bayesian approaches offer advantages of incorporating prior knowledge and transparently expressing model uncertainty to aid decision-making, they remain underused in practice. A flexible Bayesian model has recently been proposed for use in HTA settings which uses M-splines to model the hazard function, and is implemented in the survextrap R package.

**Methods:**

We conducted a simulation study to assess the statistical performance of the Bayesian survival model implemented in survextrap. We simulate survival outcomes based on control arm data from two oncology clinical trials, and generate treatment arm survival based on different realistic treatment effect scenarios. Statistical performance in modelling a single treatment arm or the difference between treatment arms is compared across a range of flexible models, varying the M-spline specification, smoothing procedure, priors, treatment effect modelling choices and other computational settings.

**Results:**

We demonstrate good model fit and convergence of complex baseline hazard functions and time-dependent covariate effects across realistic clinical trial scenarios. We show that a sufficiently flexible M-spline, implemented using a weighted random walk prior on the spline coefficients, can provide a smooth fit to the hazard without risk of overfitting, and gives unbiased estimates of restricted mean survival over the trial follow-up with good coverage of the credible intervals. Bayesian model fitting with an efficient Laplace approximation provides unbiased estimation but overestimates posterior variance. In some treatment effect scenarios, the survextrap non-proportional hazards models displayed greater bias than standard frequentist survival modelling tools such as flexsurv and rstpm2.

**Conclusions:**

This work helps inform key considerations to guide model selection and estimation performance when fitting flexible Bayesian models to trial data. These findings help identify appropriate default model settings in the software that should perform well in a broad range of settings, as well as more specific considerations to guide model selection for advanced users. This work further ensures users have greater confidence in the validity of these survival models and their implementation.

**Supplementary Information:**

The online version contains supplementary material available at 10.1186/s12874-026-02783-7.

## Background

The survextrap R package [1] has been recently developed as a flexible Bayesian approach to modelling survival outcomes from clinical trials, having particular relevance to Health Technology Assessment (HTA) where choices of parametric model (and potentially also their longer-term projections) are influential for decision making purposes. Particularly in this context, the use of flexible Bayesian survival models that use splines to model the hazard function has many potential advantages. First, the distribution of survival times and their dependence on covariates observed in clinical trials often cannot be adequately captured by standard parametric models (such as the exponential, Weibull and Gompertz models, among others), and so the use of flexible parametric models has become increasingly needed [2–9]. Bayesian methods also enable the incorporation of prior beliefs, transparent expression of model uncertainty, and facilitate the inclusion of external data (e.g., historic trial data, real-world evidence or clinical expert opinion) through evidence synthesis techniques [10–13]. Despite these potential advantages, the application of these methods in practice remains limited, and greater understanding of their statistical performance is needed.

To make effective use of flexible Bayesian survival models, there are many modelling choices that need to be considered. To this end, we performed simulation investigations with application to oncology clinical trials to compare a range of flexible models, varying the spline specification, smoothing procedure and prior specification, along with other statistical and computational modelling choices that can be chosen by the user.

While it is also important to consider how these models perform for making longer-term extrapolations, which are often influential in HTA settings, we do not explore this more specific application of the software in this work (the name “survextrap” relates to this R package design facilitating the implementation of survival extrapolation techniques). Hence our work in this study focuses on the capability of the survextrap package for generic survival modelling from a single individual-level dataset, rather than for extrapolation. Nonetheless, this current study is foundational to performing survival extrapolations, since building credible long-term projections requires an underlying model that accurately represents the observed trial data, is robust to modelling choices (spline flexibility, priors, smoothness), and is computationally stable and reliable.

In summary, in this work we seek to provide a comprehensive evaluation of a flexible Bayesian M-spline survival model, and provide recommendations for model settings that should perform well across a broad array of clinical trial scenarios (and potentially other applications where flexible Bayesian models are useful), as well as helping to identify appropriate default settings within the software. We assessed the performance of different model choices via standard frequentist measures such as bias and coverage. This work helps guide model specification and improve user-confidence in implementing a flexible Bayesian modelling approach to survival data.

## Methods

### Bayesian survival model

The survextrap R package fits a flexible parametric Bayesian survival model using M-splines to model how the hazard function changes over time. The hazard function is defined as a weighted sum of $\:n\:$basis functions [14, 15] $\:{b}_{i}(t)$ which take the form:

$$\:h(t)=\eta\sum\limits_{i=1}^{n}{p}_{i}{b}_{i}(t)$$

with scale parameter $\:\eta\:$ and basis coefficients $\:{p}_{i}$ where $\:\sum\nolimits_i{p}_{i}=1$. The basis functions are polynomials (cubic by default) and are totally determined by the knots and degree of the M-splines specified. The basis functions are restricted to be positive, so that the resulting hazard is a smooth, positive-valued function.

In the current default, survextrap uses $n=10$ basis functions that are defined on a set of knots placed at quantiles of the uncensored survival times. As the number of knots increase, the hazard becomes more flexible with potentially more turning points. In survextrap, the default number of knots is large to accommodate all plausible shapes. However, hierarchical priors protect against the risk of overfitting and help to smooth the data. This is similar to the use of frequentist penalized likelihood methods that help control the smoothness of the hazard function [16].

Priors are placed on the scale parameter $\eta$ and each of the basis coefficients $\:{p}_{i}$. A hierarchical prior on the ${p}_{i}$ is specified by defining $\mathrm{log}({p}_{i}/{p}_{1})={\gamma}_{i}$ with ${\gamma}_{1}=0$, $\:{\gamma}_{i}={\mu}_{i}+\sigma{\epsilon}_{i}$. The prior means $\:{\mu}_{i}$ are fixed and are determined to correspond to a constant hazard (see Appendix 1 for a full definition of ${\mu}_{i}$), while the parameter $\sigma$ controls the smoothness of the hazard curve, relating to the level of departure from a constant hazard. The random effects ${\epsilon}_{i}$ are specified using a weighted random walk with $\:{\epsilon}_{1}=0$ and $\:{\epsilon}_{i}\sim\mathrm{Logistic}({\epsilon}_{i-1},{w}_{i})$, where the weights ${w}_{i}$ depend on the distance between the knots (see Phillippo et al. [13] for exact definition), or alternatively by using an exchangeable model with ${\epsilon}_{i}\sim\mathrm{Logistic}(0,1)$. The default priors are $\:\eta\sim N(0,20)$, and $\sigma\sim\mathrm{Gamma}(2,1)$, though the package provides simulation-based procedures to determine priors that match a user’s substantive judgement about expected survival and hazard variations.

The Bayesian model can be extended to allow the hazard to depend on explanatory variables; the effect of these can either be constant through time with the assumption of proportional hazards, or time-varying (i.e. a non-proportional hazards model). In the proportional hazards model, the scale parameter is redefined with covariates ${\boldsymbol{\beta}}$ and explanatory variables ${{\mathbf{x}}}$:

$$\:\eta({{\mathbf{x}}})={\eta}_{0}\mathrm{exp}({\boldsymbol{\beta}}^{T}{{\mathbf{x}}})$$

In addition, a flexible non-proportional hazards model allows explanatory variables to affect the M-spline coefficients through a multinomial logistic regression where ${\gamma}_{i}({\boldsymbol{\mathrm{x}}})={\mu}_{i}+{\boldsymbol{\delta}}_{i}^{T}{\boldsymbol{\mathrm{x}}}+\sigma{\epsilon}_{i}.$ The $s$th element of the vector ${\boldsymbol{\delta}}_{i}$ describes the amount of departure from a proportional hazards model for the $s$th covariate in the region of time associated with the $\:i$th basis term. Hence, if $\:{\delta}_{is}=0$ for all $\:i$, then the $\:s$th covariate follows proportional hazards. A hierarchical prior is used for these coefficients such that ${\delta}_{is}\sim N(0,{\tau}_{s})$, which smooths the effects over the time regions. A relatively weak $\mathrm{Gamma}(\mathrm{2,1})$ non-informative prior is the default option for each of the ${\tau}_{s}$.

A number of additional features exist that the user can specify including incorporating aggregate survival data from external datasets in an evidence synthesis model, the use of cure models, modelling in an excess hazards framework, and sensitivity analyses with the waning of treatment effects in the long-term. These features are not investigated in this simulation study.

### Simulation design and data generation

A simulation study was undertaken for two different trials with distinct hazard functions, where in both cases the true hazard functions were known, allowing assessment of the frequentist properties of Bayesian flexible parametric models using survextrap. We simulated survival times from a flexible parametric Royston-Parmar model [2], which was fitted to each of the two trials.

#### Data generating mechanisms

**Fig. 1:**
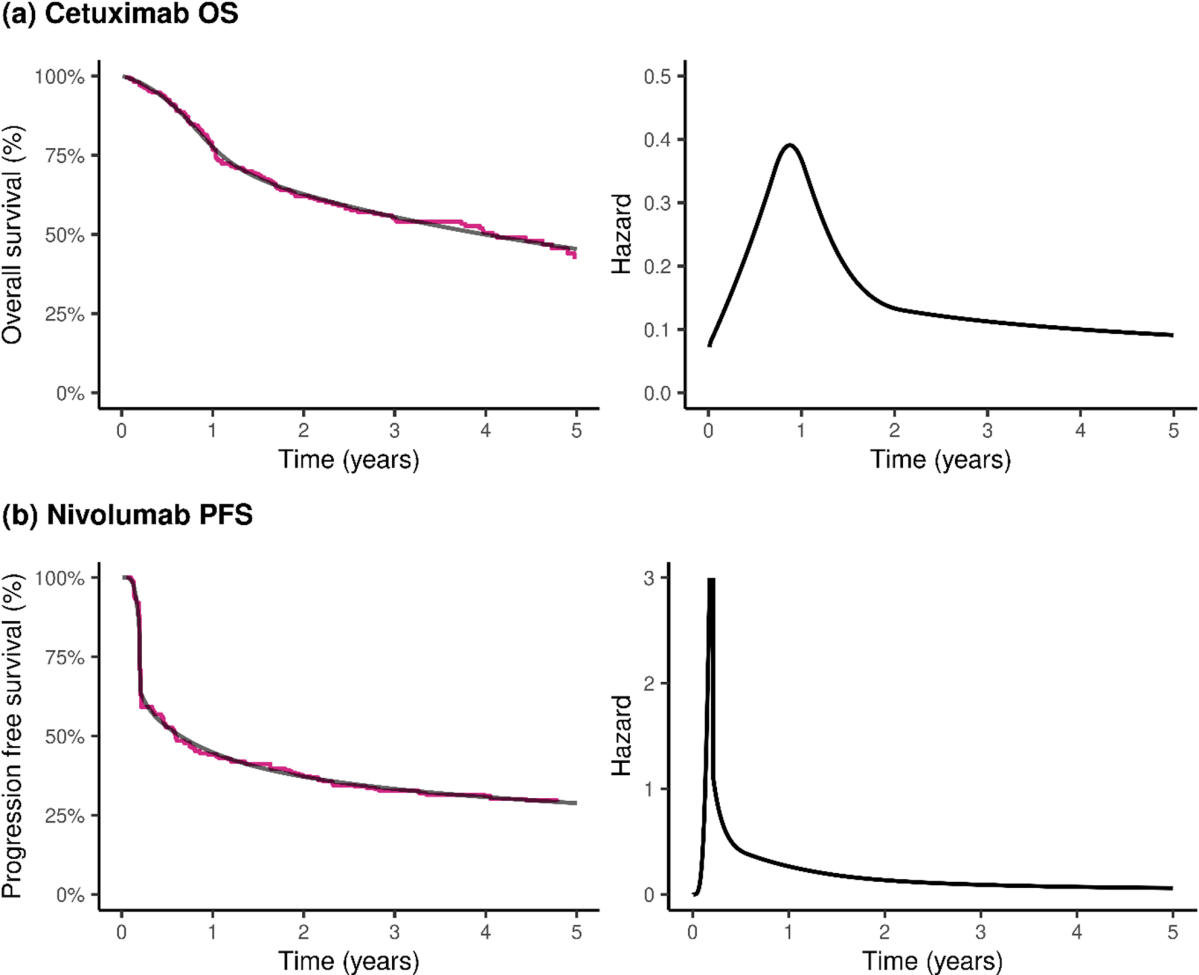
Data generating mechanisms, survival and hazard plots for the (**a**) cetuximab OS (overall survival) and (**b**) Nivolumab PFS (progression-free survival) case studies. Red line - Kaplan-Meier survival curve from trial data; black line – Royston-Parmar flexible parametric model fitted survival (left) and hazard (right) curves

For our first case study we considered overall survival for patients treated with radiotherapy plus cetuximab for head and neck cancer from Bonner et al. [17], which will be referred to as the Cetuximab OS case study hereafter. We reconstructed patient-level time-to-event data for the radiotherapy plus cetuximab arm (*N* = 211) from the Kaplan-Meier curve for overall survival using WebPlotDigitizer (version 4.5) [18] based on the algorithm by Guyot et al. [19]. We found that a Royston-Parmar spline model with 3 knots fitted in flexsurv [6] adequately captured the fit of the trial data (Fig. 1a).

As a second case study, we also analysed progression free survival for patients treated with nivolumab for advanced melanoma from Larkin et al. [20], which we refer to as the Nivolumab PFS case study, imputing patient level data from the Nivolumab PFS (*N* = 316) Kaplan-Meier curves as described above. For our true model we used a Royston-Parmar model with 6 knots fitted to Nivolumab PFS data. This represented a more widely-varying hazard function than the one derived from the cetuximab data (Fig. 1b).

For these underlying true models, we generated 1,000 single-arm datasets with a trial sample size of *N* = 200 patients. Simulated survival times were generated using the simulate function in flexsurv [6]. Administrative censoring was applied at 5 years. We also considered further scenarios corresponding to early and intermediate data cuts of the trial with shorter follow-up periods, where administrative censoring was applied at 2 and 3 years.

**Fig. 2:**
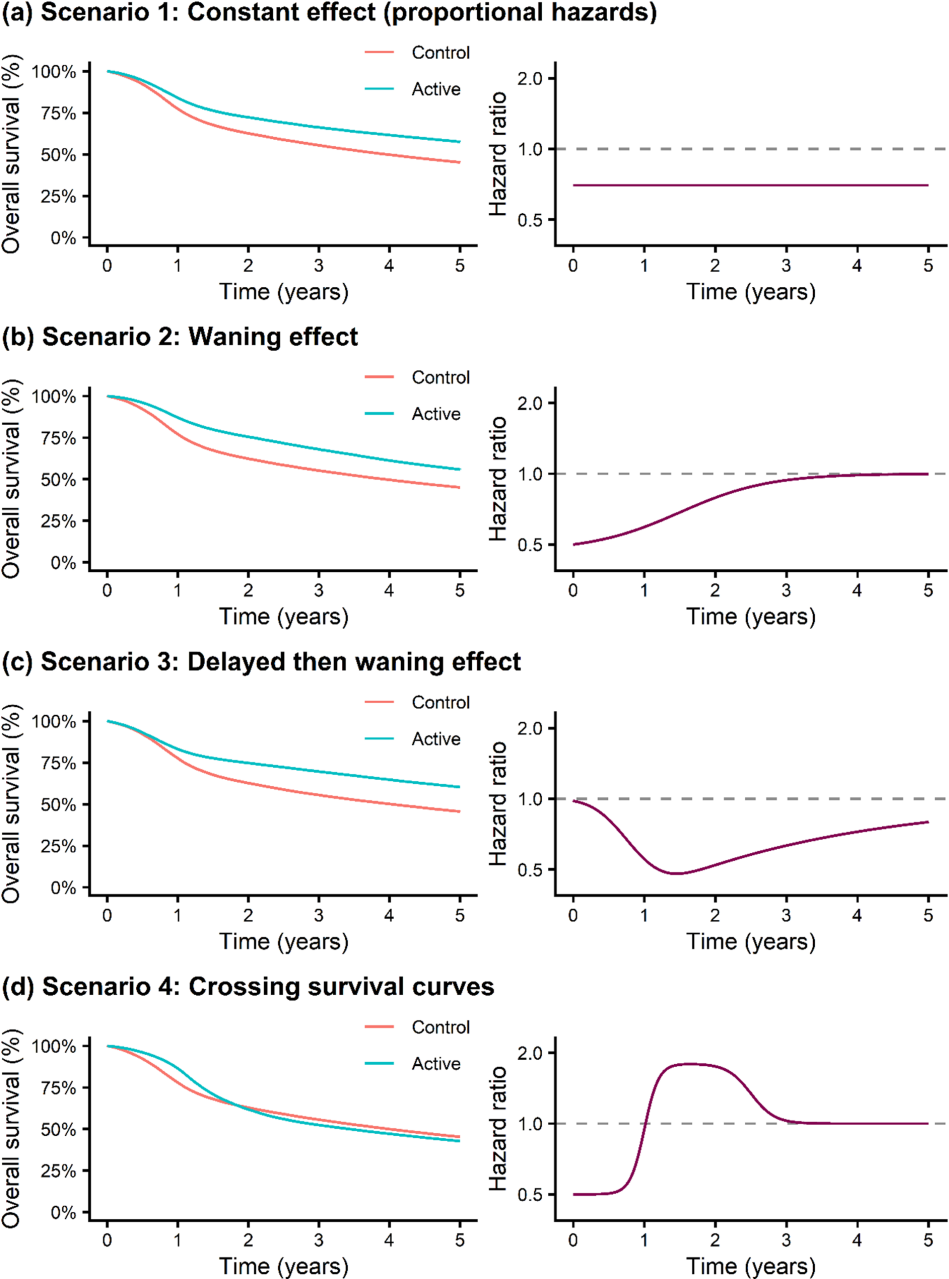
Data generating mechanisms, survival and hazard ratio plots for 4 treatment effect scenarios. Treatment effect scenarios are (**a**) Scenario 1: Constant effect (proportional hazards), (**b**) Scenario 2: Waning effect, (**c**) Scenario 3: Delayed then waning effect, (**d**) Scenario 4: Crossing survival curves

To understand the performance of survextrap for evaluating treatment effects, we used the model for radiotherapy plus cetuximab OS described above as our control arm, and further defined an active arm by specifying a hazard ratio across trial follow-up time that is relative to the cetuximab OS control arm. We consider four treatment effect scenarios; a constant treatment effect with proportional hazards (scenario one), an immediate treatment effect followed by treatment waning (scenario two), a delayed treatment effect followed by treatment waning (scenario three), and a treatment effect with crossing survival curves (scenario four). The closed-form expressions for the hazard ratio functions in each scenario are shown in Supplementary Table 1 and can be seen in Fig. 2.

To simulate event times for two arms we implemented the cumulative hazard inversion method [21] in R software. Since the cumulative hazard function for the active arm does not have a closed-form solution for these scenarios, we used numerical integration to derive the cumulative hazard using Gauss-Legendre quadrature with 100 nodes. For this two-arm setting, we generated 1,000 datasets with a trial sample of size of *N* = 400 patients, with 200 patients in each arm.

#### Estimands

Our primary estimand was the restricted mean survival time (RMST) at 5 years for the single treatment arm setting and the difference in RMST at 5 years (RMSTD) for the treatment effect between active and control. Additionally, in further trial scenarios (restricted to the single treatment arm setting) corresponding to early and intermediate data cuts with 2- and 3-years follow-up, we evaluated RMST at the 2- and 3-years landmarks, respectively. For each simulation setting we generated one very large dataset (*N* = 10^8^) without censoring and empirically calculated the RMST and RMSTD using their sample mean. We took these to be the true values for each data generating setting, which were accurate to 2 decimal places based on Monte Carlo standard errors of the sample means. Further details of the estimands and performance measures are given in Appendix 2.

#### Analysis of simulated data

Bayesian flexible parametric models were fitted to each simulated dataset using the survextrap package (version 0.8.12) [1]. We considered a range of Bayesian models, with different specifications of the cubic M-spline hazard function, while further varying the priors for the smoothing parameter $\sigma$. We varied the M-spline degrees of freedom (df) between df = 3, 6 and 10, and also varied the $\sigma\:\:$prior between Gamma(2,1), Gamma(2,5) and Gamma(2,20). To interpret these different priors for $\sigma$, we note that a Gamma(2,1) prior allows for a very flexible hazard function and gives an upper 90% credible limit for the ratio between the 90% and 10% quantiles of the hazard $h(t)$ (with respect to time) of 4.2×10^8^, while a Gamma(2,20) prior is much more restrictive and gives an upper 90% credible limit of 1.6 for this ratio. We further varied the choice of prior for the spline coefficients, evaluating models with the weighted random walk and the exchangeable model. This led to 18 combinations of model settings. A further comparison was made of the standard versus smoothed M-spline basis options, details of which are in the Appendix 3. 

For each of these 18 Bayesian models, we further varied the choice of Bayesian computation method (from the rstan package [22], that survextrap calls). Firstly, we fitted all models using the default MCMC sampling routine with 4 chains and 2,000 iterations. We additionally analysed all simulated datasets using the fast method of evaluating the posterior median using a Laplace optimisation approximation to the full posterior distribution.

Additionally, we compared the survextrap Bayesian model with frequentist spline-based approaches, where we used Royston-Parmar models implemented in flexsurv and rstpm2 R packages. These models use natural cubic splines on the cumulative hazard scale. We fitted Royston-Parmar models with 2, 3, 4, 6 and 10 df. The standard errors on RMST estimates were computed using the bootstrap method with 1,000 iterations for flexsurv and the delta method for rstpm2.

For each of the four treatment effect scenarios we assessed the performance of the survextrap non-proportional hazards models. For the non-PH models we fitted models with 3, 6 and 10 df, while varying the strength of the non-proportionality smoothness prior $\tau$ between Gamma(2,1), Gamma(2,5) and Gamma(2,20), and keeping the prior for the mean log hazard ratio at a vague Normal(0,20). For comparison, we also fitted the survextrap proportional hazards model with standard settings of df = 10, $\sigma$~Gamma(2,1) and random walk prior.

For benchmarking comparisons, we further evaluated treatment effects in both flexsurv and rstpm2. In each case we modelled the control arm using a baseline hazard with 2, 3, 4, 6 and 10 df. For the time-varying effect in flexsurv, treatment covariates were placed on each spline coefficient as ancillary parameters, so the model was fully stratified by treatment, with the time-varying treatment effect having the same form of spline as the baseline hazard (same degree, knot locations and basis functions with different coefficients). In rstpm2 there is the additionally flexibility with the tvc argument to specify time-varying effects with a spline of a different form to the baseline hazard, and we varied the spline for the time-varying treatment effect between 1, 2, 3 and 6 df while varying the baseline hazard df as described above.

## Results

### Estimating the RMST for a single treatment arm

#### Cetuximab OS case study

For the cetuximab OS case study, the true survival was 62.7%, 55.5% and 45.4% at 2, 3 and 5-year timepoints, respectively, while the true restricted mean survival (RMST) at 5 years was 3.19 years (Fig. 1a).

**Fig. 3:**
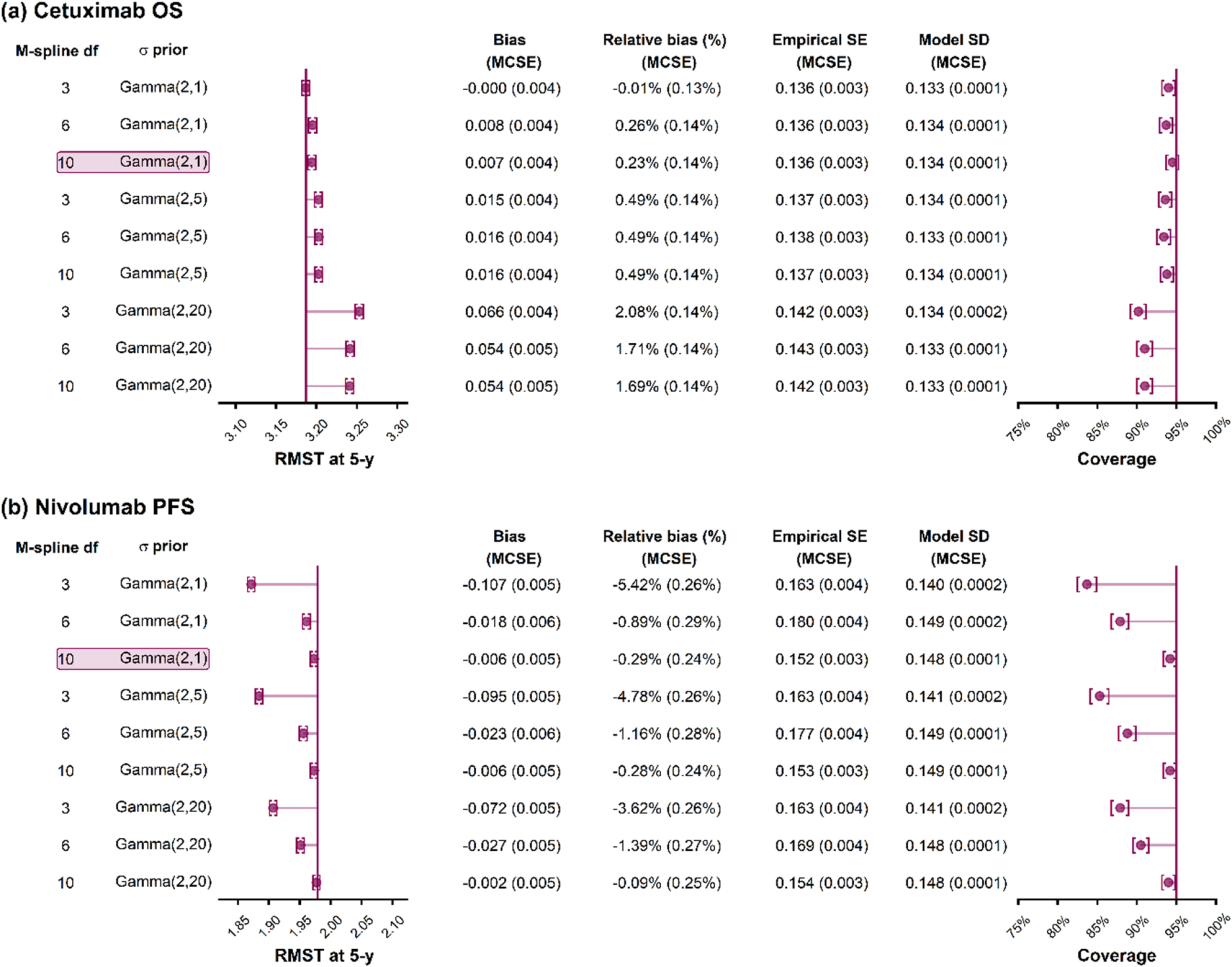
Performance measures for the posterior median RMST at 5-y based on simulated data from the (**a**) cetuximab OS and (**b**) nivolumab PFS case studies using a random walk prior for survextrap. Highlighted rows show the default settings in survextrap

Across 1,000 simulated datasets with 200 patients, the average number of events within 5-year follow-up time was 113 (s.d. = 7). The performance measures for estimating 5-y RMST for cetuximab OS are shown in Fig. 3a. For the survextrap model using default options of 10 df for the M-spline, a smoothness prior $\sigma$~Gamma(2,1) and a random walk prior on the spline coefficients, the mean estimate of 5-y RMST across 1,000 simulations was 3.19 (Monte Carlo standard error (MCSE) = 0.004), which corresponds to an absolute bias of 0.007 (0.004) years and relative bias 0.23% (0.14%). For this model, the posterior standard deviation of 5-y RMST, averaged across simulations, was 0.134 (0.0001), which matched the true sampling variability, measured by the empirical standard error of 0.136 (0.003). The coverage of the 95% credible intervals for this model was 94.5% (0.7%). The fitted survival and hazard curves were centred around the true curve and had the same shape (Supplementary Fig. 1).

In addition, for this model we have also provided samples from the prior distribution of the hazard, the M-spline weights ${p}_{i}$, and the prior trajectories of the individual M-spline basis contributions ${p}_{i}{b}_{i}(t)$, all in Supplementary Fig. 2. As expected, the prior hazard function trajectories are highly flexible and sharply changing in terms of turning points, with wide variations in the potential contributions of each M-spline term across prior draws. 

We also observed comparable model fit when varying the df of the M-spline hazard function whilst keeping the default smoothing prior of Gamma(2,1) (Fig. 3a), with low absolute and relative bias (< 1%), and only slight undercoverage. When increasing the strength of the smoothness prior $\sigma$ to Gamma(2,5) and Gamma(2,20), we observed increases in bias and poorer coverage (Fig. 3a). The prior distributions for these models with stronger smoothness priors are shown in Supplementary Figs. 3 and 4, where we can see the flatter hazard trajectories, as well as more even weighting of the M-splines over time. We found that these stronger priors only achieved sufficient shrinkage when using a weighted random walk prior on the spline coefficients (Supplementary Fig. 1), while in contrast, there was minimal shrinkage towards a constant hazard with the exchangeable model (Supplementary Fig. 5). Nevertheless, the exchangeable model gave comparable performance to the random-walk model in terms of bias across the scenarios investigated (Supplementary Fig. 6).

**Fig. 4:**
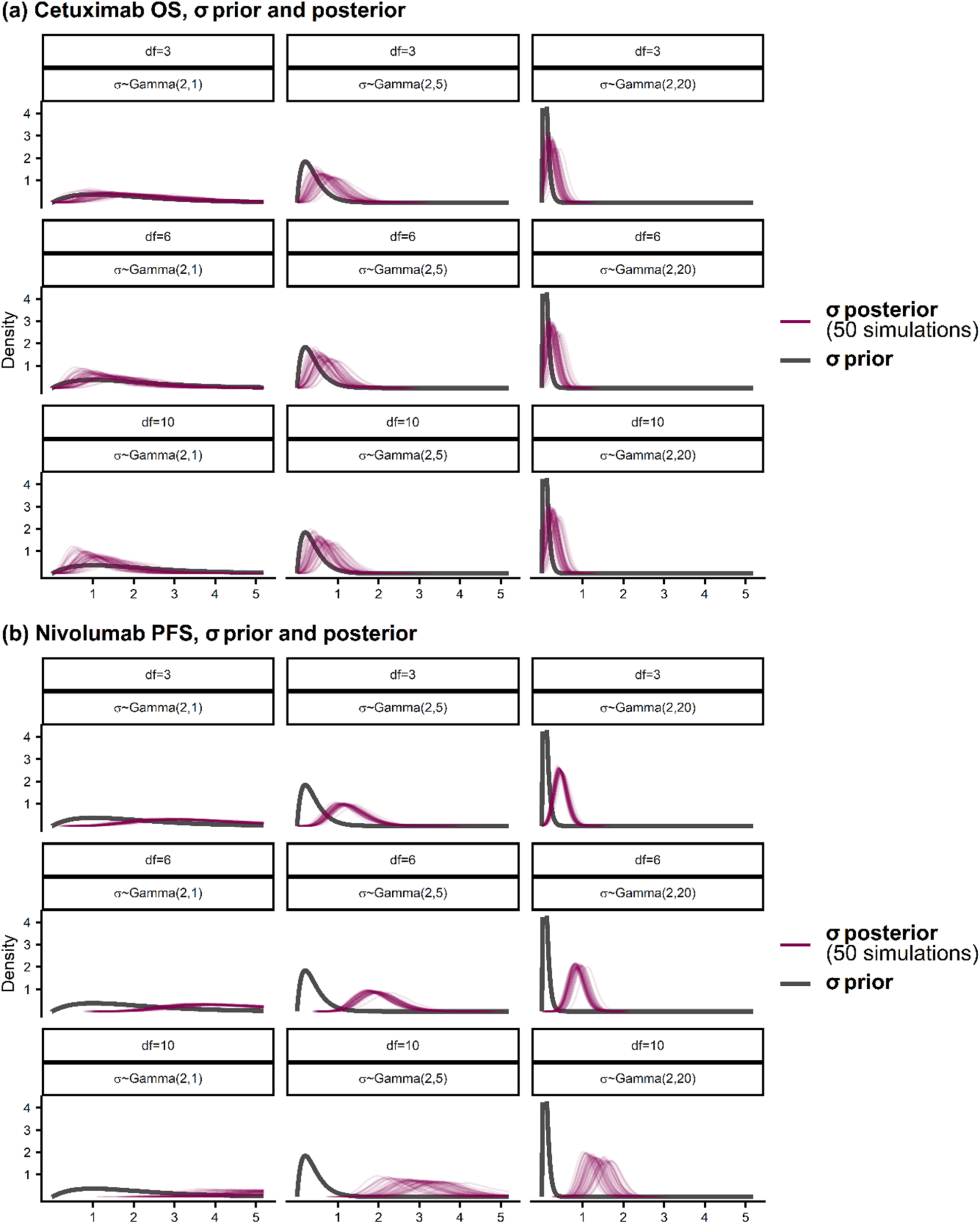
Prior and posterior distributions of the smoothness parameter $\sigma$ across scenarios for (**a**) cetuximab OS and (**b**) nivolumab PFS, with a random walk prior on the spline coefficients. Posterior is shown for models fitted from 50 simulated datasets

For cetuximab OS the performance was sensitive to the choice of $\sigma$ prior. To further investigate this, we compared the prior and posterior distribution of $\sigma$ across modelling scenarios, with the posterior distribution shown for 50 simulated datasets (Fig. 4). We found close overlap between the prior and posterior distributions (Fig. 4a), indicating reasonable prior-data agreement. This leads to the prior being influential and having an observable regularising effect on the smoothness of the predicted hazard (Supplementary Fig. 1). Comparisons of the prior and posterior distributions for $\sigma$ were also broadly unchanged between the random walk and exchangeable spline prior models (Supplementary Fig. 7). We further assessed the MCMC convergence performance of the survextrap models (Supplementary Fig. 8). With df = 10, $\sigma$ ~ Gamma(2,1) and a random walk prior, we found that 0.1% of simulation replicates had poor convergence as indicated by $\widehat{R}>$ 1.05. We observed that 29% of the 1,000 simulation replicates reported at least one divergent transition in the MCMC sampler, and among the chains with at least one divergent transition, the proportion of the total iterations that diverged remained low at 0.5%. As such, any divergences in the MCMC sampler were minor and did not appear to adversely impact estimation performance. For this model we also found reasonable MCMC sampling efficiency across the posterior, with only 1.7% of simulation replicates having low bulk effective sample size (< 400, where the combined chains had length 8,000). Convergence was better using the random walk prior compared to the exchangeable prior (Supplementary Fig. 9).

#### Nivolumab PFS case study

For the Nivolumab PFS case study the true 5-y RMST was 1.98 years (Fig. 1b), and the true PFS survival probability at 5 years was 28.8%. A survextrap model with default settings (df = 10, $\sigma$ ~ Gamma(2,1) and a random walk prior) gave a mean estimate of 5-y RMST of 1.97 (0.005) years, with minimal absolute bias of −0.006 (0.005) years and relative bias −0.29% (0.24%), and reasonable coverage probability of 94.2% (0.8%) (Fig. 3b). This model had sufficient flexibility to adequately capture the sharp hazard function and steep gradient of the true survival curve (Supplementary Fig. 10). 

When varying the model parameters, we noted that for a model with an M-spline hazard function with 3 df, there was marked downward bias in estimates of 5-y RMST (Fig. 3b), as this model did not capture the sharp gradient present in the true survival curve (Supplementary Figs. 10 and 11). Hence, for nivolumab PFS the results were sensitive to the M-spline df.

We note also that performance was less influenced by the $\sigma$ prior in the nivolumab PFS case study as compared to the cetuximab OS case study. To examine this, we note for nivolumab PFS there was sharper disagreement between the prior and posterior distributions of $\sigma$ (Fig. 4b). This indicates the data (e.g., likelihood) being more dominant than the prior in the posterior, which is due to both a higher number of events than in the cetuximab OS case study, as well as a sharper observed hazard that requires less shrinkage for an adequate fit, leading to a right-shifted posterior distribution for $\sigma$. As such, the performance was less dependent on the choice of $\sigma$ prior. 

#### Fast Laplace approximation

We assessed the Laplace approximation method within survextrap as a fast approximation to full MCMC sampling, with results presented in Supplementary Fig. 12. The runtime for the Laplace optimisation approximation was ~ 0.4 s per model fit compared to ~ 50 s using full MCMC, for models with 10 df (Supplementary Table 2). All models fitted using the optimisation approximation converged. For all models tested and for both case studies, we observed that the optimisation approximation had point estimates comparable to the full MCMC sampler, with similar bias. However, the model posterior standard deviations were higher than for models estimated using full MCMC, resulting in over coverage of 97.0% (0.5%) under the default settings (df = 10, $\:\sigma$ ~ Gamma(2,1)).

#### Early and intermediate data cuts

We investigated scenarios with shorter follow-up of 2- and 3-years (Supplementary Fig. 13). For cetuximab OS, survival was immature at these landmarks with median survival not yet reached. Bias was low, with absolute bias < 0.05 years and relative bias under 1.5%, with good coverage. Slight improvement in estimation was shown under models with 6 and 10 df (compared with 3 df), as well as in models using the less informative ${\upsigma}$ ~ Gamma(2,1) prior. For nivolumab PFS, the default settings of a model with 10 df and ${\upsigma}$ ~ Gamma(2,1) resulted in negligible bias in RMST at the respective landmark times, but slight undercoverage of 93.0% (0.81%) and 94.5% (0.72%) at 2 and 3-years, respectively.

#### Comparison with standard frequentist models

Performance measures for estimates of 5-y RMST using Royston-Parmar spline approaches, implemented in both flexsurv and rstpm2 R packages, are shown in Supplementary Fig. 14. In comparison to the survextrap model with default settings, we found that Royston-Parmar models with sufficient flexibility had comparable performance when estimating 5-y RMST. For the cetuximab OS case study, we noted that Royston-Parmar models with at least 3 df performed well, with very low bias (relative bias < 0.9%), whilst for the nivolumab PFS case study the best performance was achieved for models with at least 4 df (relative bias < 1.1%). We noted that Royston-Parmar models with df = 2, equivalent to a standard parametric Weibull model, had relative bias of 3.8% (0.13%) and 6.7% (0.25%) for the cetuximab OS and nivolumab PFS case studies, respectively, highlighting the dangers with using an under-parameterised parametric model. For the models with sufficient df, the model standard errors were well-calibrated.

### Estimating the difference in RMST between two treatment arms

**Fig. 5:**
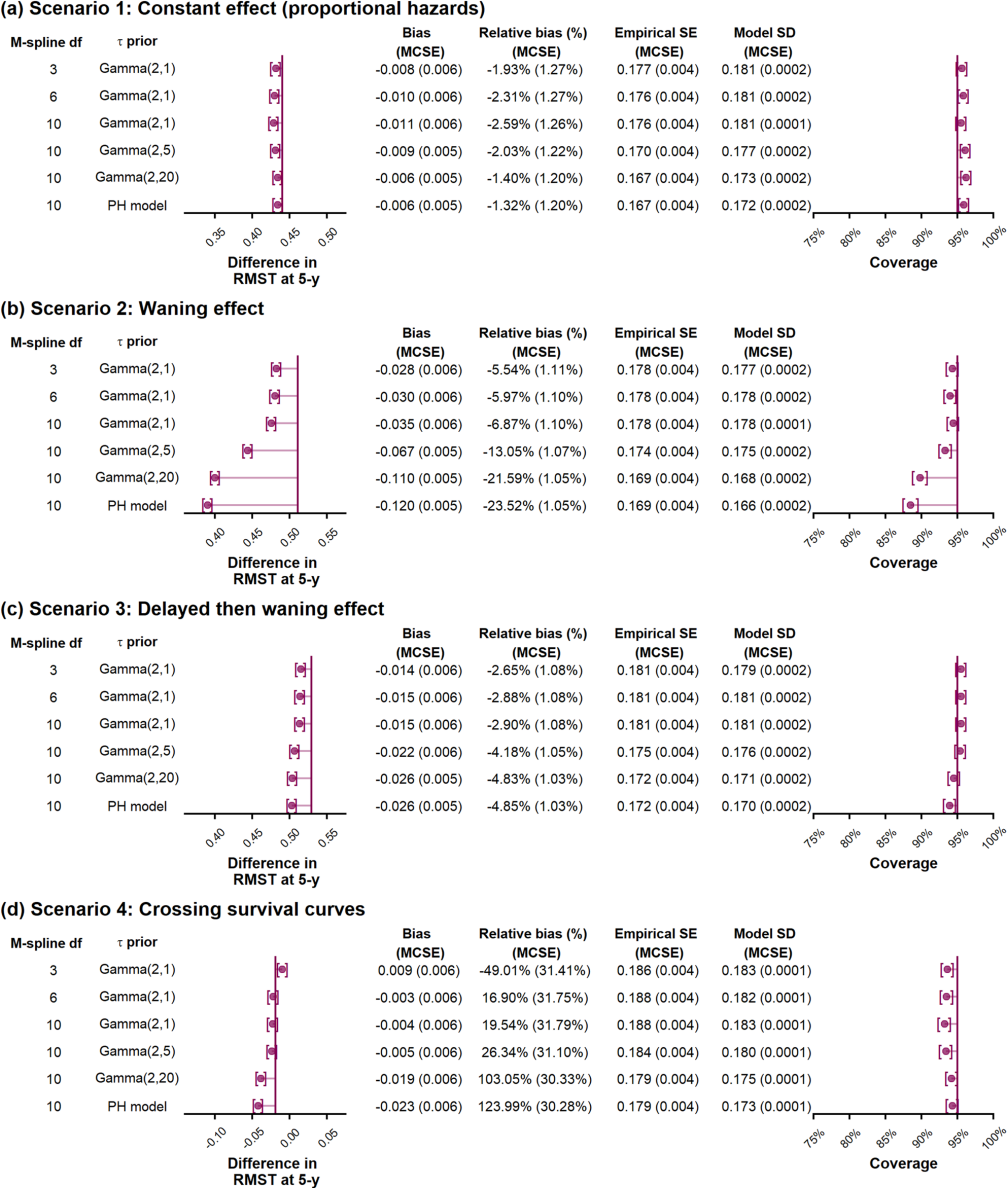
Performance measures for the difference in RMST at 5-y based on simulated data from the cetuximab OS case study for the control arm and under four scenarios for the time-varying treatment effect, investigating fitted survextrap models that use a random walk prior and a default Gamma(2,1) prior for $\:\sigma\:$ and where the df and prior for $\tau$ are varied. Treatment effect scenarios are (**a**) Scenario 1: Constant effect (proportional hazards), (**b**) Scenario 2: Waning effect, (**c**) Scenario 3: Delayed then waning effect, (**d**) Scenario 4: Crossing survival curves

For the four treatment effect scenarios (Fig. 2) the true difference between treatment arms in restricted mean survival time (RMSTD) at 5 years for scenario one (constant treatment effect, proportional hazards), two (waning treatment effect), three (delayed treatment effect followed by waning) and four (crossing survival curves) was 0.44, 0.51, 0.53 and −0.02 years, respectively. The performance of survextrap in estimating RMSTD is shown in Fig. 5, where all models have a random walk prior on the spline coefficients and with a Gamma(2,1) prior for $\sigma$.

For scenario one, where the true underlying treatment effect was constant with hazard ratio (HR) = 0.7, we found that all survextrap models estimated this treatment effect reasonably well. For the survextrap proportional hazards (PH) model (df = 10) the mean 5-y RMSTD was 0.43 (0.006) years, corresponding to a bias of −0.006 (0.005) years and relative bias −1.32% (1.20%). The survextrap non-PH model with df = 10 and non-proportionality smoothing prior $\tau$ ~ Gamma(2,1) showed slight underestimation of the treatment effect with bias of −0.011 (0.006) years and relative bias −2.59% (1.26%). For the survextrap non-PH models, we found that strengthening the non-proportionality prior on $\tau$ to Gamma(2,5) and Gamma(2,20) lead to similar estimates to the PH model in this scenario (Fig. 5a), as expected.

For scenarios two (waning treatment effect) and three (delayed treatment effect followed by waning), where the underlying true models had time-varying treatment effects (Fig. 2b and c), we observed improved estimation performance for survextrap non-PH models with fewer df (df = 3 or 6) compared with the default setting of df = 10 (Fig. 5b and c). For scenario two (waning treatment effect), the survextrap non-PH model with df = 10, $\tau$ ~ Gamma(2,1) had more notable bias of −0.035 (0.006) years and relative bias −6.87% (1.10%), whilst under a more simplified non-PH model (df = 3, $\tau$ ~ Gamma(2,1)), the absolute bias reduced to −0.028 (0.006) years with relative bias of −5.54% (1.11%), though bias was still evident. A similar pattern was found for scenario three (delayed treatment effect followed by waning), with the non-PH model with df = 10, $\tau$ ~ Gamma(2,1) having a relative bias of −2.90% (1.08%), while for a less complex model (df = 3, $\tau$ ~ Gamma(2,1)), the bias remained but was slightly reduced −2.65% (1.08%). 

For scenario four (crossing survival curves, Fig. 2d), absolute bias was −0.004 (0.006) years using the default setting of df = 10, $\tau$ ~ Gamma(2,1). Relative bias appeared high due to the true RMSTD (the denominator) being close to zero, but for the default setting the bias was within MCSE of zero. For both the PH model and a non-PH model with stronger smoothing on the non-proportionality effect, $\tau$ ~ Gamma(2,20), the lack of flexibility led to bias in 5-y RMST becoming more apparent.

**Fig. 6:**
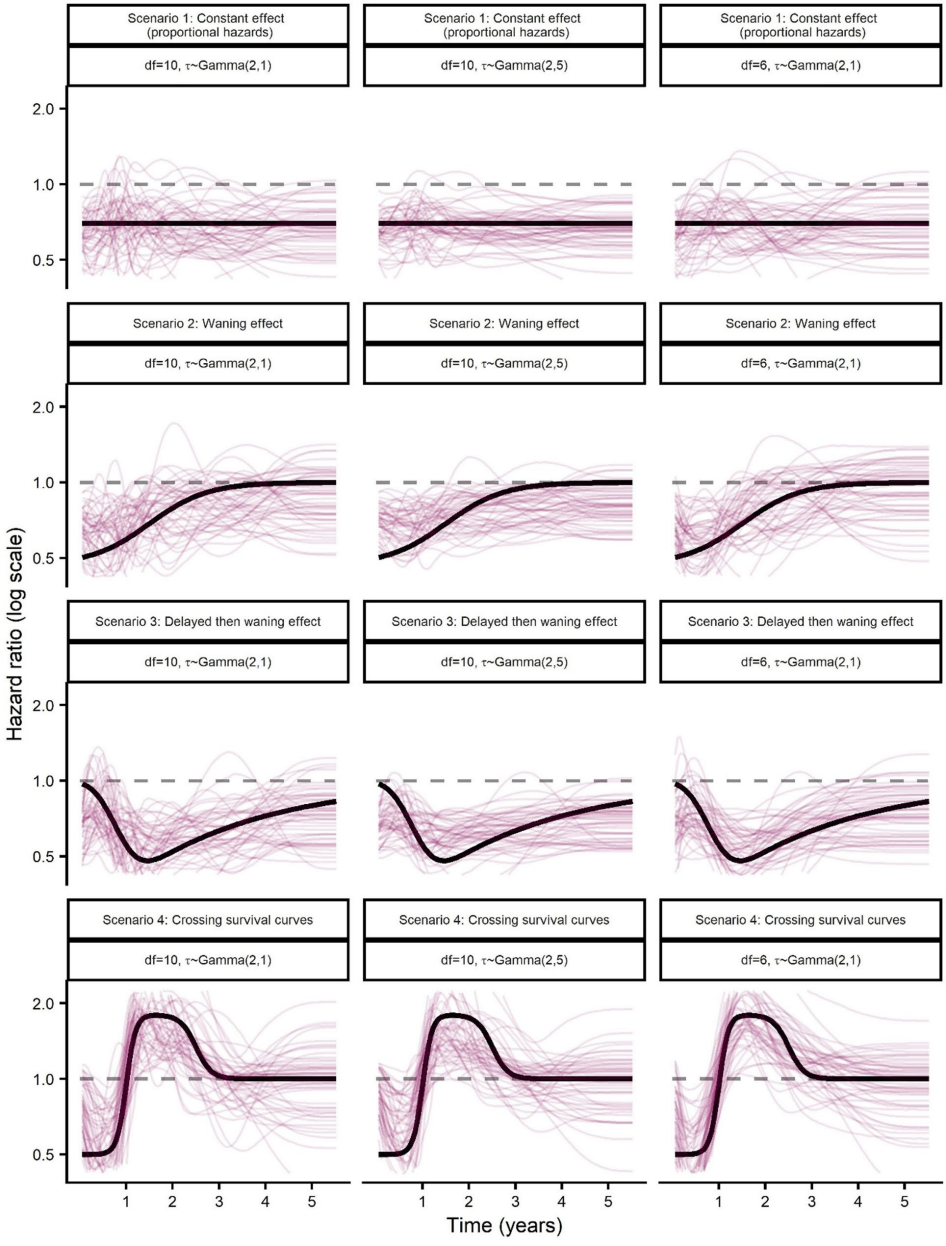
Hazard ratio plots for a non-proportional hazards survextrap model fitted using a random walk prior and varying the df and non-proportionality smoothness prior $\tau$, based on 50 simulated datasets under four treatment-effect scenarios

The visual fit of the estimated hazard ratio for a random sample of 50 simulation replicates is shown in Fig. 6. We note that the non-PH models with a Gamma(2,1) prior for $\tau$ show reasonable closeness of fit to the underlying true hazard ratio, though power is perhaps limited at this trial sample size. We note that strengthening the smoothing on the amount of non-proportionality by changing the prior on $\tau$ from Gamma(2,1) to Gamma(2,5) flattened out the estimated time-varying treatment effects, leading to underestimation of the 5-y RMSTD in Scenarios 2 and 3 (Fig. 5), suggesting this flattening was excessive. Additionally, when fitting models with an exchangeable prior on the spline coefficients (Supplementary Figs. 15 and 16), we observed a higher number of turning points and broad divergences from the true treatment effect over the trial period suggestive of overfitting. Model runtime was higher for the non-proportional hazards models and models with higher df (Supplementary Table 3).

#### Comparison with standard frequentist models

Performance measures for treatment effects using frequentist Royston-Parmar spline approaches implemented in flexsurv and rstpm2 are shown in Supplementary Fig. 17. For scenario one (constant treatment effect), models with 2 df (equivalent to a Weibull standard parametric model) displayed slight bias, while all other models demonstrated unbiased estimation (within MCSE) of 5-y RMSTD. For scenario two (waning treatment effect), we found only slight bias for all models with at least 3 df on the time-varying effect and at least 4 df on the baseline spline, which reflected improved performance over the survextrap models. Similarly, in scenario three (delayed treatment effect followed by waning) and scenario four (crossing survival curves), we noted that unbiased estimates (within MCSE) were achieved for more flexible models with at least 4 df for both the baseline hazard and time-varying effect. In scenario three, model and empirical standard errors were closely aligned, leading to good coverage at the 95% level for the models with sufficient df. In scenario four, we noted slight undercoverage across all models considered.

## Discussion

In this study, we have sought to understand the performance of the survextrap R package for flexible Bayesian modelling of survival data through a simulation approach applied to case examples from oncology clinical trials.

A primary aim was to help provide users with an understanding of which model specifications best capture the survivor functions through the presentation of the bias and coverage of the estimated RMST at 5-years, and the difference in RMST between two treatment arms. We compared a range of models with different choices for the flexibility of the baseline hazard function, which is dictated both by the number of knots in the M-spline and the prior distributions for the coefficients of the spline. A summary of recommendations, along with strengths and limitations of the survextrap R package for survival modelling are presented in Table 1.

**Table 1:** Strengths, limitations and practical recommendations for using the survextrap R package for survival modelling of clinical trial data (this does not include considerations specific to extrapolation, which is a topic of future work)

Strengths	Limitations	Practical Recommendations
• The flexible Bayesian M-spline model can capture complex hazard shapes and non-proportional hazards, while the prior structure ensures the fitted hazard is smooth. • Fast Laplace approximations provide computationally efficient and broadly unbiased point estimates with an approximate uncertainty distribution, useful for model exploration. • Clarity of user interface, with single survextrap command for model fitting, and single commands used for posterior summaries (e.g., survival, hazard, rmst). Hence the user is not required to perform lower-level Bayesian implementation (e.g., Stan) or bespoke coding to extract posterior estimates. • Supports models used for extrapolation in HTA, including incorporation of real-world data through evidence synthesis modelling, along with cure and relative survival models.	• While simulations have shown the defaults are reasonable in a typical situation, the models include a lot of parameters and settings that may need to be tuned for optimal fit in some situations. • Accurate uncertainty quantification using Hamiltonian Monte Carlo (MCMC) can be time-consuming, especially for high df and non-PH models. • The survextrap non-PH models may not be able to achieve as good a balance between flexibility and overfitting, compared to frequentist survival modelling tools such as flexsurv and rstpm2. • Stronger priors than the default settings (or a lower df) may be needed to improve MCMC convergence, particularly for the smoothness parameter $\sigma$.	• A starting point for trial-based modelling is to begin with survextrap defaults (10 df, $\sigma$ ~ Gamma(2,1) and random walk prior), and proportional hazards when plausible. This has strong and flexible estimation performance across a range of realistic scenarios from oncology trial settings. • If changing the flexibility from the default, ensure the models fit the data. • We recommend checking fit to the data with Kaplan-Meier curves and hazard plots, checking plausibility of priors (e.g. the variability in hazards implied) fully documenting modelling choices, and presenting diagnostics to assess convergence. • Use non-proportional hazards models with particular care. • Plan for computational burden in real-world applications. Use Laplace approximation for exploratory analysis, full MCMC for final inference.

As a starting point for model selection, we identified that a model with 10 degrees of freedom, a smoothness prior of $\sigma$ ~ Gamma(2,1), and a weighted random walk prior on the spline coefficients, demonstrated consistently good performance across all scenarios considered. Specifically, for estimating mean survival (5-y RMST) in a single arm, the relative bias was very low (< 1%) and the flexibility in the M-spline was sufficient to capture sharply varying hazard functions without evidence of overfitting, while achieving good computation convergence of the MCMC chains. While the default of 10 knots may seem a lot, in some situations (e.g. the nivolumab case study) such flexibility is required to capture the true hazard. The smoothness parameter $\sigma$ ensures that even if the true hazard is less flexible than this (e.g. the cetuximab case study) the data are not overfitted with 10 knots.

For estimating the difference in mean survival times across two arms, the relative bias was also quite low at ~5% across scenarios. This model specification should provide a useful set of default settings for survextrap, as some users may need to rely on these. Though we suggest that users should check the fit of their model to their own data, and consider whether fit might be improved with other choices. Alternative modelling choices, can be assessed formally using a cross-validatory procedure [23].

As part of this investigation we evaluated the performance of a weighted random walk prior on the M-spline coefficients, which is a modelling approach proposed recently by Phillippo et al. [13] adapted from Li and Cao [24], that has been newly implemented in survextrap. We found the weighted random walk prior achieved effective smoothness of the hazard, while in contrast, using an exchangeable random effect prior did not provide sufficient shrinkage toward a constant hazard, consistent with previous findings [13].

We also considered the choice of computational method that survextrap uses for implementing Bayesian estimation. The default Markov Chain Monte Carlo procedure based on Hamiltonian Monte Carlo (HMC, from the Stan software [25]) can be computationally demanding, in particular for larger datasets and models with many predictors. The package provides (also via Stan) some fast approximate Bayesian inference procedures, but the accuracy of these approximations has not been thoroughly assessed for these models. We compared the accuracy of estimates from the full MCMC procedure with a fast Laplace approximation based on determining the posterior mode by optimisation. While the fast approximation method gave good point estimates, uncertainties were not as well calibrated as the MCMC approach, especially when model flexibility was poorly specified. We also found the HMC sampler had good convergence properties in realistic trial datasets. A pragmatic recommendation could be to use the Laplace approximation method for model exploration, considering goodness of fit and leave-one-out cross-validation (LOOCV) [23], and then applying the more intensive full MCMC for final analysis.

We also provided a detailed analysis of time-dependent effects, considering a range of scenarios inspired from oncology trials. We found non-proportional hazards (non-PH) models gave broadly good estimation, even if the underlying truth was proportional hazards, though there was some evidence of poorer fit under a treatment effect waning scenario. We further showed that in a scenario with crossing survival curves, where the true underlying hazard ratio was sharply changing over time (potentially reflective of treatments with very contrasting mechanisms of action, or altogether different modalities such as radiotherapy and surgery), that the model could accurately capture more complex survival dynamics. We noted however that frequentist Royston-Parmar models (natural cubic splines) did outperform survextrap in some non-PH settings. The best survextrap model tended to depend slightly on the setting, as we found potentially fewer degrees of freedom were required for modelling treatment effects. We suggest that model selection should be performed that considers a range of models with different priors, flexibilities, including both PH and non-PH models.

We have not comprehensively investigated all modelling choices, such as the knot locations, which were left data-driven based on event quantiles, along with the degree of M-spline, which was set as cubic throughout. Nonetheless, previous studies [26, 27] using M-spline basis functions for survival modelling and other applications have shown robustness to these specifications. Note also that the Bayesian models investigated in this paper used the package’s default, weakly informative priors. If strong priors are used that conflict with the truth, these may result in bias.

In keeping with the design of survextrap, we suggest that further work should evaluate the modelling performance for longer-term projections of survival. This requires broader modelling considerations, such as structural assumptions in the tail, assessments of clinical plausibility, integration of external data (e.g., elicited expert opinion, real-world data, population mortality rates), scenario analyses (treatment waning and cure), and downstream health-economic implementation (ICERs, PSA, state-transition models), all of which are beyond the scope of this current study. Thus, the broader topic of extrapolation performance is the subject of further ongoing research on these models.

## Conclusions

Our simulation results demonstrate the flexible Bayesian survival model implemented in the survextrap R package has good performance across a range of realistic scenarios, correctly identifying complex baseline hazards and time-dependent covariate effects. We additionally provide comprehensive assessment of many modelling features such as the appropriate model flexibility, the impact of choices of prior and spline specification, the convergence of MCMC sampling routines, and the performance of more computationally efficient Laplace approximation settings. This work helps inform key considerations to guide model selection and estimation performance when fitting flexible Bayesian models to trial data, as well as helping to identify appropriate default model settings in the software that should perform well in a broad range of settings, especially those relevant to clinical trial and HTA contexts. In summary, our work helps further provide users with an understanding of the key modelling choices, and ensures greater confidence in the validity of these survival models and their implementation in the survextrap software.

## Supplementary Information

Supplementary Material 1.

## Data Availability

All data and code that support the findings of this study are available at the following URL: github.com/irtimmins/Simulation-study-survextrap.
