# Supplementary material for: Simulation-based assessment of a Bayesian M-spline survival model with flexible baseline hazard and time-dependent effects
Source: BMC Med Res Methodol. 2026 Feb 12;26:60. doi: 10.1186/s12874-026-02783-7 (PMC12997853; doi:10.1186/s12874-026-02783-7)
Supplement: Supplementary file 1 — Supplementary Material 1. [file 12874_2026_2783_MOESM1_ESM.pdf]

Supplementary Information: Simulation-based assessment of a Bayesian M-spline survival model with flexible baseline hazard and time-dependent effects

Contents

Appendix 1: Further details on the M-spline construction and priors 6

Appendix 2: Estimands and performance measures 8

Appendix 3: Standard and smoothed M-spline basis functions 10

Supplementary Table 1: Treatment effect functions for each of the four simulation scenarios. 11

Supplementary Table 2: Stan model fitting mean run times for single arm case studies, comparing full MCMC with the Laplace optimisation 'opt' approximation (N = 200). 12

Supplementary Table 3: Stan model fitting mean run times for two-arm treatment effect scenarios, comparing full MCMC with the Laplace optimisation 'opt' approximation, all with N = 400 patients (200 in each arm). 13

Supplementary Figure 1: Survival and hazard plots for a `survextrap` model fitted using a random walk prior on the spline coefficients based on 50 simulated datasets from the cetuximab OS case study. 15

Supplementary Figure 2: Prior hazard function trajectories, M-spline weights p_i, and the prior trajectories of the individual M-spline basis contributions $p_i b_i(t)$ for df = 10 and $\sigma \sim$ Gamma(2,1). The y-axis of the hazard curves has been slightly curtailed to enable more direct comparison across Supplementary Figure 2, 3 and 4. 16

Supplementary Figure 3: Prior hazard function trajectories, M-spline weights p_i, and the prior trajectories of the individual M-spline basis contributions $p_i b_i(t)$ for df = 10 and $\sigma \sim$ Gamma(2,5). The y-axis of the hazard curves in this figure has been

slightly curtailed to enable more direct comparison across Supplementary Figure 2, 3 and 4. 17

Supplementary Figure 4: Prior hazard function trajectories, M-spline weights p_i, and the prior trajectories of the individual M-spline basis contributions $p_i b_i(t)$, for df = 10 and $\sigma \sim$ Gamma(2,20). 18

Supplementary Figure 5: Survival and hazard plots for a `survextrap` model fitted using an exchangeable prior on the spline coefficients based on 50 simulated datasets from the cetuximab OS case study. 19

Supplementary Figure 6: Performance measures for the posterior median RMST at 5-y based on simulated data from the cetuximab OS and nivolumab PFS case studies using an exchangeable prior for `survextrap`. 20

Supplementary Figure 7: Prior and posterior distributions of the smoothness parameter σ across scenarios for cetuximab OS and nivolumab PFS, with an exchangeable prior on the spline coefficients. Posterior is shown for models fitted from 50 simulated datasets. 21

Supplementary Figure 8: Stan warning messages and convergence diagnostics for modelling a single treatment arm using a random walk prior on the spline coefficients. 22

Supplementary Figure 9: Stan warning messages and convergence diagnostics for modelling a single treatment arm using an exchangeable prior on the spline coefficients. 23

Supplementary Figure 10: Survival and hazard plots for a `survextrap` model fitted using a random walk prior on the spline coefficients based on 50 simulated datasets from the Nivolumab PFS case study. 24

Supplementary Figure 11: Survival and hazard plots for a `survextrap` model fitted using an exchangeable prior on the spline coefficients based on 50 simulated datasets from the Nivolumab PFS case study. 25

Supplementary Figure 12: Performance measures for the posterior median RMST at 5-y based on simulated data from the Cetuximab OS and Nivolumab PFS case studies using a Laplace approximation method (`fit_method = "opt"`) for `survextrap` and a random walk prior. 26

Supplementary Figure 13: Performance measures for the posterior median RMST at 2- and 3-y based on simulated data from the Cetuximab OS and Nivolumab PFS case studies with shorter follow-up of 2 and 3 years, respectively. Models use a random walk prior. Highlighted rows show the default settings in `survextrap`. 27

Supplementary Figure 14: Performance measures for the RMST at 5-y based on simulated data from the Cetuximab OS and Nivolumab PFS case studies using frequentist models implemented in R packages `flexsurv` and `rstpm2`. 28

Supplementary Figure 15: Performance measures for the difference in RMST at 5-y based on simulated data from the Cetuximab OS case study for the control arm and under four scenarios for the time-varying treatment effect, investigating fitted `survextrap` models that use an exchangeable prior and a default Gamma(2,1) prior for σ and where the degrees of freedom and prior for τ are varied. 29

Supplementary Figure 16: Hazard ratio plots for a non-proportional hazards `survextrap` model fitted using an exchangeable prior and varying the degrees of freedom and non-proportionality smoothness prior τ, based on 50 simulated datasets under four treatment-effect scenarios. 30

Supplementary Figure 17: Performance measures of frequentist methods for estimating the difference in RMST at 5-y (Scenario 4 overleaf). 31

Supplementary Figure 18: Performance measures for estimating 5-y RMST using the standard M-spline basis functions (unsmoothed at the upper boundary knot, option `bsmooth = FALSE`) and smoothed basis functions (smoothed at upper boundary knot, option `bsmooth = TRUE`). Models fitted using a random walk prior. 33

References 34

Appendix 1: Further details on the M-spline construction and priors

The hazard function is modelled as

$$h(t) = \eta \sum_{i=1}^{n} p_i b_i(t)$$

where $\eta > 0$ and p_i: $i = 1, \ldots, n$, $\sum_i p_i = 1$ are parameters to be estimated, and the basis functions $b_i(t)$ are deterministic functions of time t.

Construction from knot sequence

To construct M-spline basis functions $b_i(t)$ of order k (i.e., for cubic M-splines set $k = 4$) on a finite interval $[L, U]$, define a non-decreasing knot sequence $t_1 \leq t_2 \leq \cdots \leq t_{n+k}$ where

- $t_1 = \cdots = t_k = L$, the lower boundary knot
- t_{k+1} up to t_n comprise the internal knots
- $t_{n+1} = \cdots = t_{n+k} = U$, the upper boundary knot

By default, the lower boundary knot is set to $L = 0$, and the internal knots and upper boundary knots are set as the quantiles of event times in the survival data.

The basis functions are defined recursively. For $k = 1$,

$$b_i(t|k=1) = \begin{cases} 1/(t_{i+1} - t_i) & \text{if } t_i \leq t < t_{i+1} \\ 0 & \text{otherwise} \end{cases}$$

Then for each subsequent order k, $b_i(t|k)$ are defined in terms of the basis functions of the previous order $k - 1$:

$$b_i(t|k) = \frac{k[(t - t_i)b_i(t|k-1) + (t_{i+k} - t)b_{i+1}(t|k-1)]}{(k-1)(t_{i+k} - t_i)}$$

The basis functions are positive in (t_i, t_{i+k}) and zero elsewhere, and integrate to 1 on the interval $[L, U]$.

A closely related family of splines called B-splines are defined as

$$B_i(t|k) = \frac{(t_{i+k} - t_i)b_i(t|k)}{k}$$

These have the alternative normalisation of $\sum_i B_i(t) = 1$ for all t. Using this result, if we take $p_i = (t_{i+k} - t_i)/[k(U - L)]$ for $i = 1, \ldots, n$, then the M-spline $\sum_i p_i\, b_i(t)$ has the property of being constant with time. This property is used to define the constant hazard prior below.

Priors

To define the `survextrap` model, priors are placed on the scale parameter η and each of the basis coefficients p_i. A hierarchical prior on the p_i is specified by defining $\log(p_i/p_1) = \gamma_i$ with $\gamma_1 = 0$, $\gamma_i = \mu_i + \sigma\epsilon_i$. The prior means μ_i are fixed and are determined to correspond to a constant hazard (see above for the related discussion of M-splines and B-splines), and can thus be expressed in terms of the knots as:

$$\mu_i = \log\left(\frac{t_{i+k} - t_i}{t_{k+1} - t_1}\right)$$

The random effects ϵ_i are specified using a weighted random walk with $\epsilon_1 = 0$ and $\epsilon_i \sim$ Logistic(ϵ_{i-1}, w_i), where the weights w_i depend on the distance between the knots (see Phillippo et al. [1] for exact definition), or alternatively by using an exchangeable model with $\epsilon_i \sim$ Logistic$(0,1)$. The default priors are $\eta \sim N(0, 20)$, and $\sigma \sim$ Gamma(2,1).

Appendix 2: Estimands and performance measures

We evaluated the frequentist properties of the different models, considering the bias, empirical standard errors and model-based posterior standard deviation, and coverage, while reporting the Monte Carlo standard errors for each (see Morris et al. [2] for full definitions of these performance measures). These measures were computed using the `rsimsum` R package [3]. For the single-arm cases we compared estimates to the true values obtained from the underlying parametric model derived in `flexsurv`. We further plotted the fitted survival and hazard function for a random sample of 50 simulation replicates, to evaluate the similarity of their shapes (over time) to the true hazard and survival.

For the treatment-effect, we evaluated the difference in RMST at 5-y (RMSTD) as our estimand. Since the true value cannot be computed using a closed-form expression we instead evaluated the sample estimate of 5-y RMSTD from a very large simulated data set ($N = 10^8$) and took this to be the truth (accurate to 2 decimal places). We also further visually assessed the hazard ratio estimates from the `survextrap` non-PH models against the true values.

Finally, for the models fitted using the full MCMC sampler we assessed how the choice of Bayesian model impacted computational stability using a range of diagnostics. For each model, we first evaluated the proportion of the 1,000 simulations for which "divergent transitions", arising from problems with the Hamiltonian Monte Carlo procedure, were reported by `rstan`, or any other `rstan` warning message. We further assessed whether the models displayed high $\hat{R}$ values (>1.05), which are suggestive of poor mixing or convergence of the MCMC procedure. We also identified models with low effective sample size (bulk or tail ESS <400), which measure sampler

efficiency and reliability of the Monte Carlo estimates of the posterior quantiles from the sample. For models fitted using the Laplace optimisation approximation method, we computed the proportion of simulation iterates that converged to the posterior mode. We further assessed the mean run-time for each `survextrap` model fit.

Appendix 3: Standard and smoothed M-spline basis functions

M-spline functions were originally designed to only model data within a pair of "boundary" knots [4]. However, parametric survival modelling, and extrapolation in particular, requires a hazard function $h(t)$ to be defined at all times t. Therefore, the `survextrap` model modifies the original M-spline specification [4] to assume a constant hazard after the final boundary. By default, for consistency with typical spline modelling practice, an additional smoothness constraint is defined by setting the derivative and second derivative of $h(t)$ at the upper boundary to be zero (see Appendix C from Jackson [5]). However, it is unclear whether this constraint is beneficial for modelling data in practice.

To address this, analyses were conducted where we evaluated models using the standard (without boundary constraint) and smoothed (with boundary constraint) set of M-spline basis functions, specified using the `bsmooth` argument. As the standard option requires at least 4 degrees of freedom, we only compared these models for df = 6 and 10.

When estimating 5-y RMST for cetuximab OS using a `survextrap` model with df = 10 and $\sigma \sim$ Gamma(2,1), the estimation performance was comparable across both choices of M-spline basis, with relative bias 0.23% (0.14%) and 0.19% (0.14%) for a smoothed and standard basis, respectively. Applying these models to Nivolumab PFS, the relative bias using the standard basis was slightly higher at 0.80% (0.25%) compared to the default smoothed basis at -0.29% (0.24%) (Supplementary Figure 18). For both cetuximab OS and nivolumab PFS case studies, as we varied both the M-spline degrees of freedom and strength of the σ smoothing prior, we found no material difference in model performance for estimating 5-y RMST.

Supplementary Table 1: Treatment effect functions for each of the four simulation scenarios.

Scenario	Hazard ratios comparing the active relative to control arm
1	$HR(t) = 0.7$
2	$HR(t) = -0.38 + 0.38\tanh(0.8t - 1.2)$<br>where $\tanh(x) = \frac{e^x + e^{-x}}{e^x - e^{-x}}$ is the hyperbolic tangent function.
3	$HR(t) = -2.8\, f_{emg}(t;\ \mu = 0.8\,, \sigma = 0.4, \lambda = 0.35)$<br>where f_{emg} is the probability density function of an exponentially modified Gaussian distribution:<br>$f_{emg}(x;\ \mu\,, \sigma, \lambda) = \frac{\lambda}{2} e^{\frac{\lambda}{2}(2\mu + \lambda - 2x)} \mathrm{erfc}\left(\frac{\mu + \lambda\sigma^2 - x}{\sqrt{2}\sigma}\right)$<br>And erfc is the complementary error function:<br>$\mathrm{erfc}(x)\text{=1-}\mathrm{erf}(x) = \frac{2}{\sqrt{\pi}} \int_x^{\infty} e^{-s^2}\, ds$
4	$HR(t) = \exp\left[\left(\alpha + (\beta - \alpha)s(t)\right)d(t)\right]$<br>With $s(t)$ based on the hyperbolic tangent:<br>$s(t) = \gamma(1 + \tanh(\delta(t - \tau_1))$<br>And $d(t)$ a logistic curve:<br>$d(t) = 1 - \frac{1}{1 + \exp\left(-\varepsilon(t - \tau_2)\right)}$<br>Where $\alpha = \log(0.5)$, $\beta = \log(1.8)$, $\gamma = 0.5$, $\delta = 5, \varepsilon = 6$, and $\tau_1 = 1$, $\tau_2 = 2.5$.

Supplementary Table 2: Stan model fitting mean run times for single arm case studies, comparing full MCMC with the Laplace optimisation 'opt' approximation (N = 200).

Case Study	Time (seconds)	Stan method	M-spline df
**Cetuximab OS**	31.8	MCMC	3
	40.2	MCMC	6
	49.7	MCMC	10
	0.22	Opt	3
	0.30	Opt	6
	0.41	Opt	10
**Nivolumab PFS**	30.3	MCMC	3
	58.0	MCMC	6
	92.8	MCMC	10
	0.22	Opt	3
	0.30	Opt	6
	0.42	Opt	10

Supplementary Table 3: Stan model fitting mean run times for two-arm treatment effect scenarios, comparing full MCMC with the Laplace optimisation 'opt' approximation, all with N = 400 patients (200 in each arm).

Scenario	Time (seconds)	Stan method	Model	M-spline df
**Proportional hazards**	110.2	MCMC	PH	10
	187.8	MCMC	Non-PH	3
	200.0	MCMC	Non-PH	6
	249.3	MCMC	Non-PH	10
	1.23	Opt	PH	10
	0.89	Opt	Non-PH	3
	1.21	Opt	Non-PH	6
	1.60	Opt	Non-PH	10
**Treatment waning**	105.7	MCMC	PH	10
	205.1	MCMC	Non-PH	3
	217.0	MCMC	Non-PH	6
	251.6	MCMC	Non-PH	10
	1.13	Opt	PH	10
	0.83	Opt	Non-PH	3
	1.07	Opt	Non-PH	6
	1.47	Opt	Non-PH	10
**Treatment delay then waning**	106.9	MCMC	PH	10
	164.3	MCMC	Non-PH	3
	199.6	MCMC	Non-PH	6
	249.7	MCMC	Non-PH	10
	1.33	Opt	PH	10
	0.88	Opt	Non-PH	3
	1.12	Opt	Non-PH	6
	1.56	Opt	Non-PH	10
**Crossing survival curves**	103.2	MCMC	PH	10
	165.0	MCMC	Non-PH	3
	167.6	MCMC	Non-PH	6
	229.7	MCMC	Non-PH	10
	1.39	Opt	PH	10
	0.63	Opt	Non-PH	3
	0.88	Opt	Non-PH	6

	1.41	Opt	Non-PH	10

Supplementary Figure 1: Survival and hazard plots for a `survextrap` model fitted using a random walk prior on the spline coefficients based on 50 simulated datasets from the cetuximab OS case study.

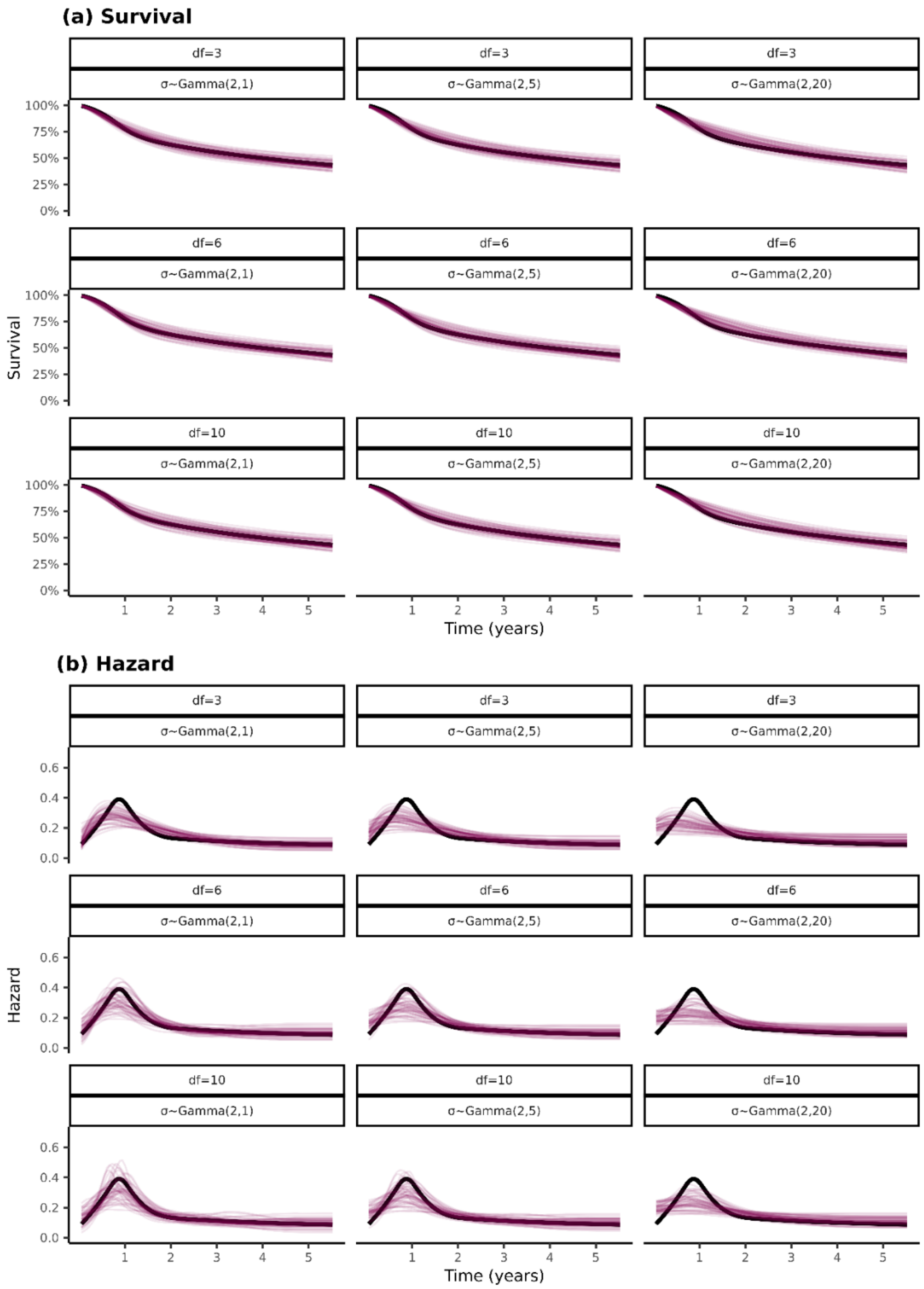

The black line shows the true survival and hazard function, the maroon lines show the model estimates.

Supplementary Figure 2: Prior hazard function trajectories, M-spline weights $\boldsymbol{p_i}$, and the prior trajectories of the individual M-spline basis contributions $\boldsymbol{p_i b_i(t)}$ for df = 10 and $\boldsymbol{\sigma} \sim \text{Gamma}(2,1)$. The y-axis of the hazard curves has been slightly curtailed to enable more direct comparison across Supplementary Figure 2, 3 and 4.

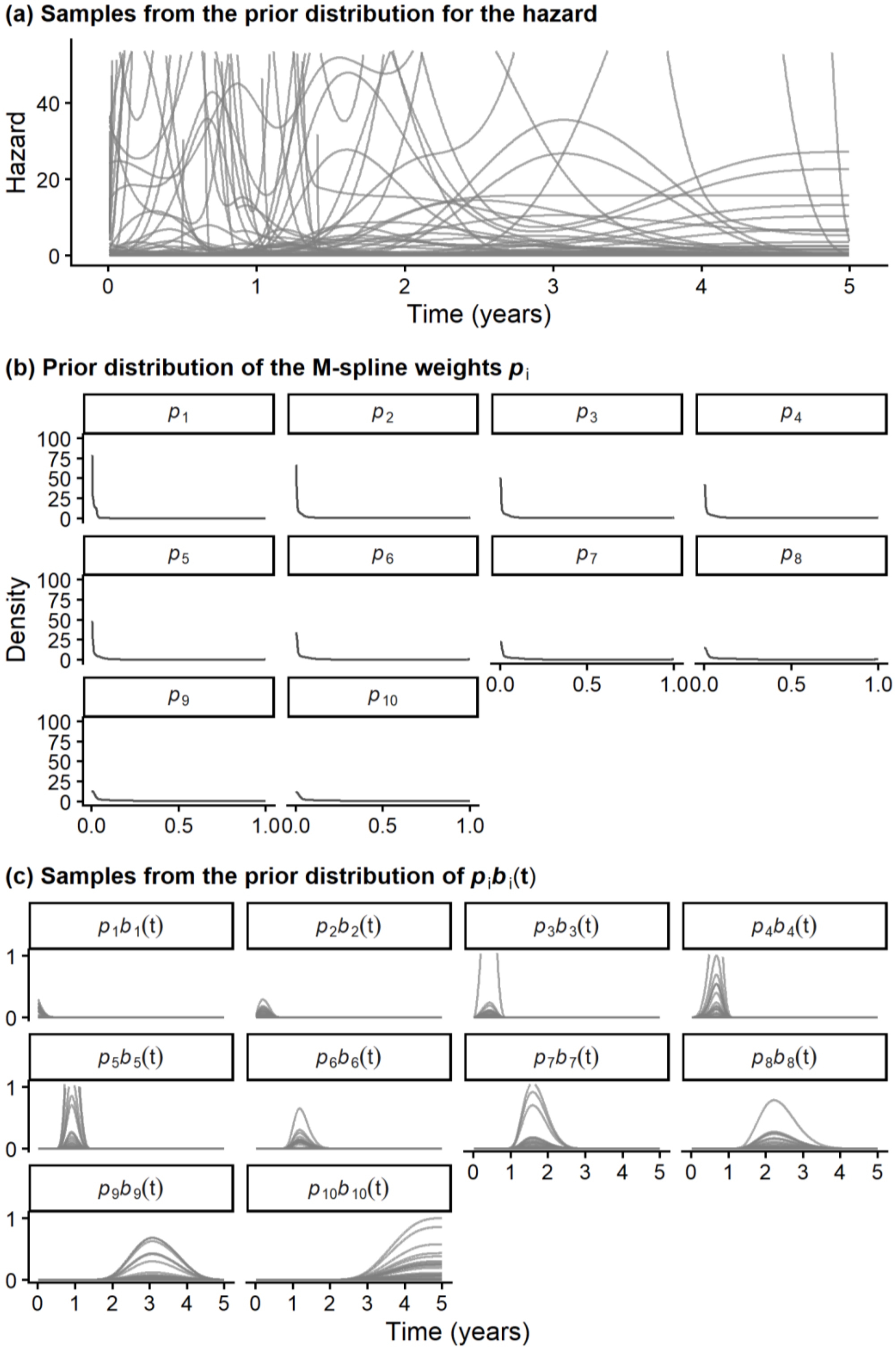

Supplementary Figure 3: Prior hazard function trajectories, M-spline weights $\boldsymbol{p_i}$, and the prior trajectories of the individual M-spline basis contributions $\boldsymbol{p_i b_i(t)}$ for df = 10 and $\boldsymbol{\sigma} \sim$ Gamma(2,5). The y-axis of the hazard curves in this figure has been slightly curtailed to enable more direct comparison across Supplementary Figure 2, 3 and 4.

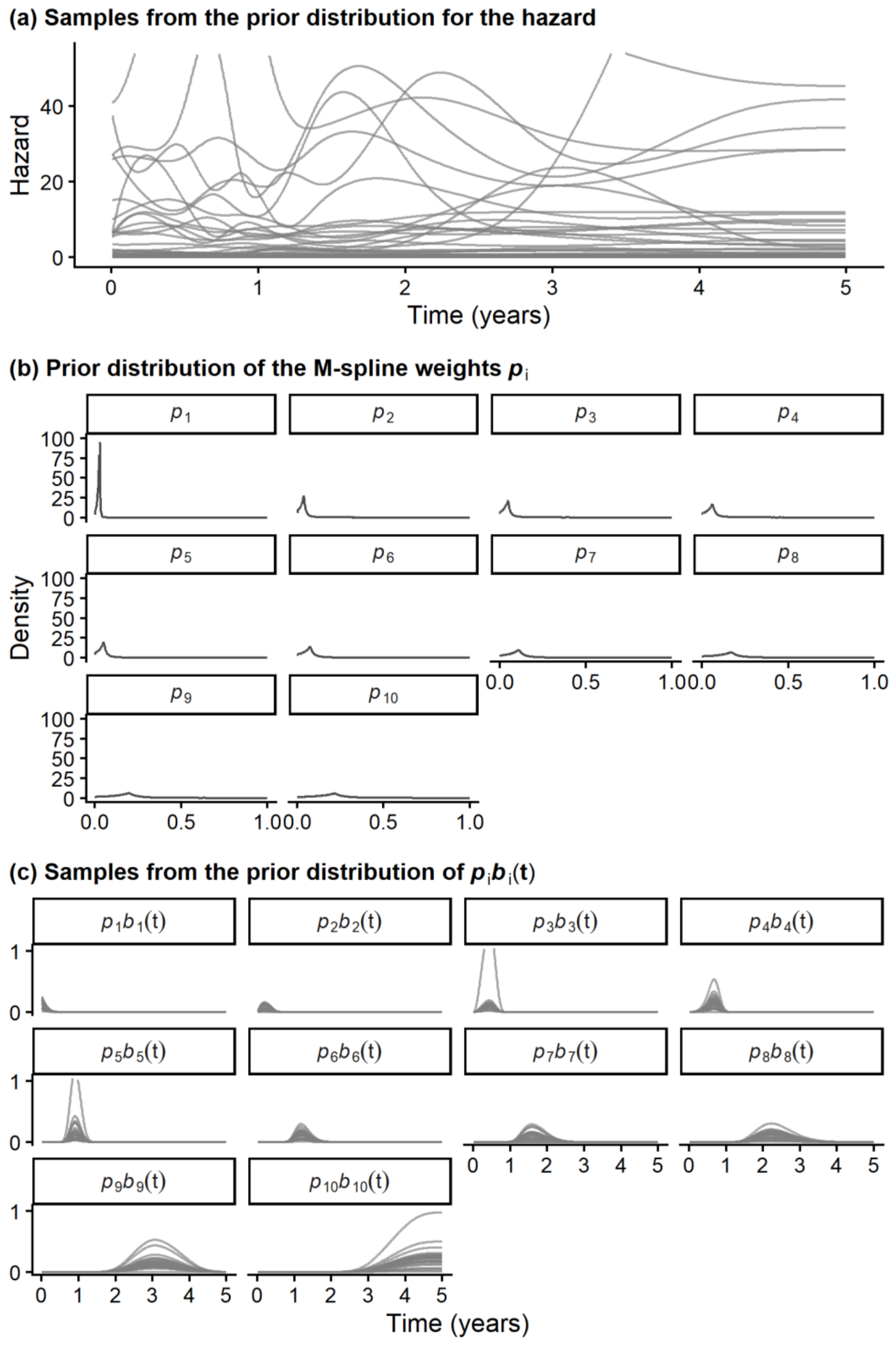

Supplementary Figure 4: Prior hazard function trajectories, M-spline weights $\boldsymbol{p_i}$, and the prior trajectories of the individual M-spline basis contributions $\boldsymbol{p_i b_i(t)}$, for df = 10 and $\boldsymbol{\sigma} \sim$ Gamma(2,20).

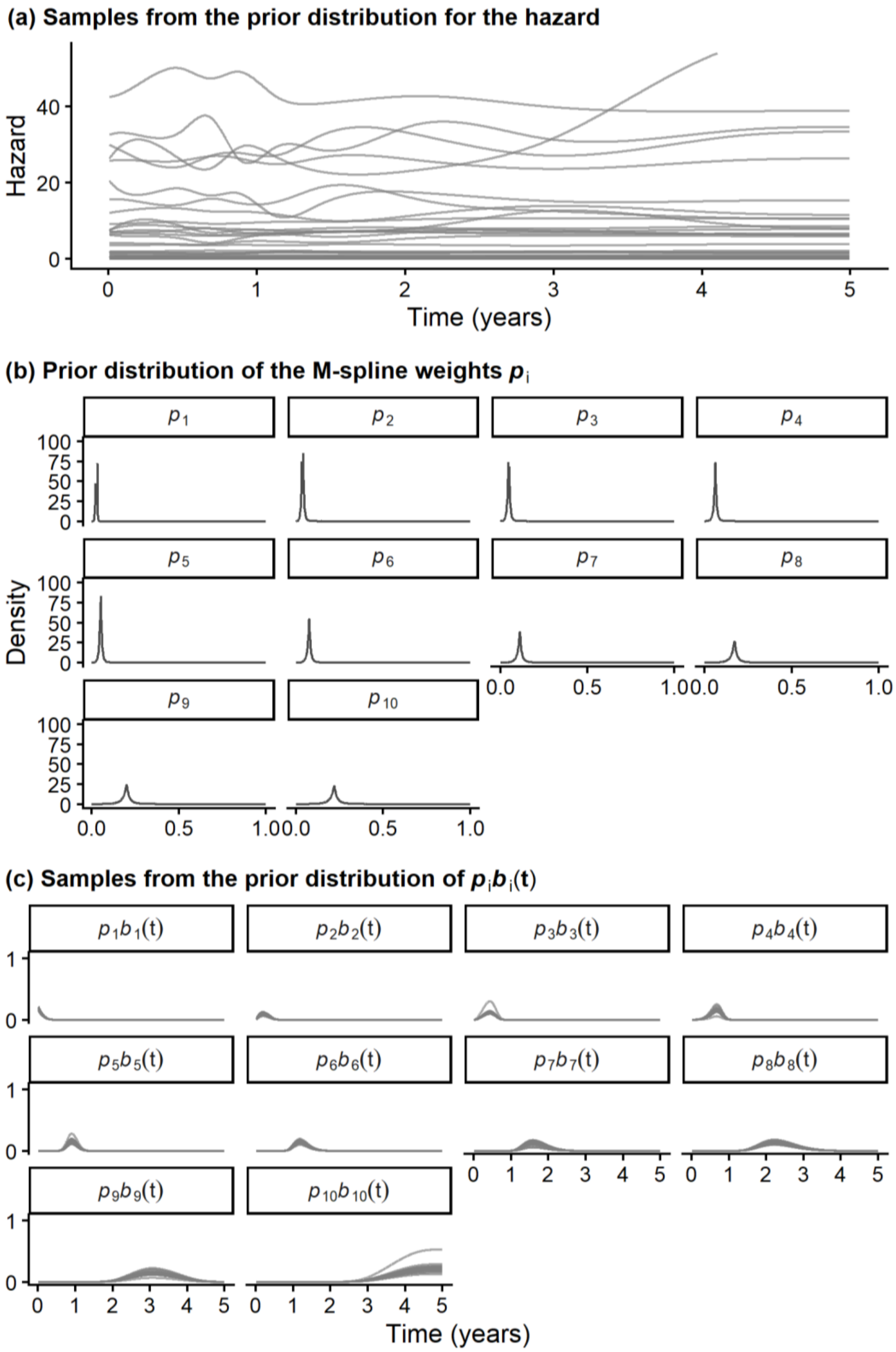

Supplementary Figure 5: Survival and hazard plots for a `survextrap` model fitted using an exchangeable prior on the spline coefficients based on 50 simulated datasets from the cetuximab OS case study.

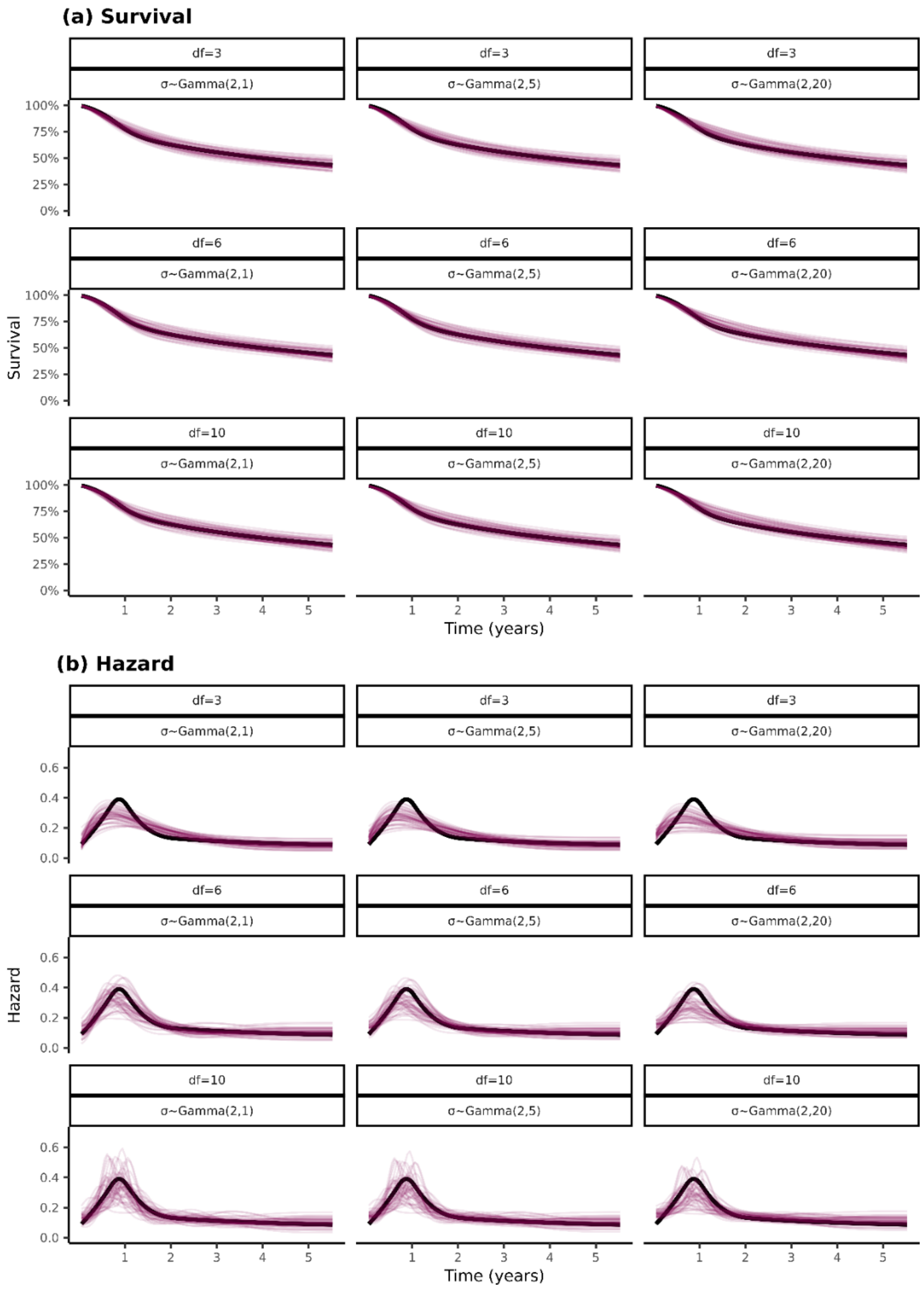

The black line shows the true survival and hazard function, the maroon lines show the model estimates.

Supplementary Figure 6: Performance measures for the posterior median RMST at 5-y based on simulated data from the cetuximab OS and nivolumab PFS case studies using an exchangeable prior for `survextrap`.

(a) Cetuximab OS

M-spline df	σ prior	Bias (MCSE)	Relative bias (%) (MCSE)	Empirical SE (MCSE)	Model SD (MCSE)
3	Gamma(2,1)	-0.005 (0.004)	-0.16% (0.13%)	0.136 (0.003)	0.133 (0.0001)
6	Gamma(2,1)	0.011 (0.004)	0.34% (0.13%)	0.136 (0.003)	0.134 (0.0001)
10	Gamma(2,1)	0.015 (0.004)	0.47% (0.14%)	0.136 (0.003)	0.133 (0.0001)
3	Gamma(2,5)	0.001 (0.004)	0.04% (0.14%)	0.136 (0.003)	0.133 (0.0001)
6	Gamma(2,5)	0.016 (0.004)	0.50% (0.13%)	0.136 (0.003)	0.133 (0.0001)
10	Gamma(2,5)	0.023 (0.004)	0.73% (0.14%)	0.137 (0.003)	0.133 (0.0001)
3	Gamma(2,20)	0.027 (0.004)	0.83% (0.14%)	0.140 (0.003)	0.133 (0.0001)
6	Gamma(2,20)	0.036 (0.004)	1.14% (0.14%)	0.139 (0.003)	0.132 (0.0001)
10	Gamma(2,20)	0.048 (0.004)	1.51% (0.14%)	0.140 (0.003)	0.132 (0.0001)

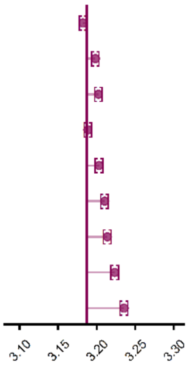

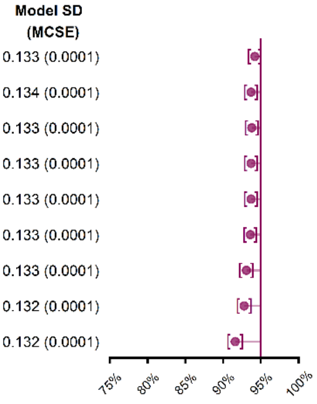

(b) Nivolumab PFS

M-spline df	σ prior	Bias (MCSE)	Relative bias (%) (MCSE)	Empirical SE (MCSE)	Model SD (MCSE)
3	Gamma(2,1)	-0.111 (0.005)	-5.62% (0.26%)	0.163 (0.004)	0.140 (0.0002)
6	Gamma(2,1)	-0.010 (0.006)	-0.53% (0.29%)	0.184 (0.004)	0.149 (0.0002)
10	Gamma(2,1)	-0.001 (0.005)	-0.07% (0.24%)	0.152 (0.003)	0.148 (0.0001)
3	Gamma(2,5)	-0.107 (0.005)	-5.40% (0.26%)	0.163 (0.004)	0.140 (0.0002)
6	Gamma(2,5)	-0.007 (0.006)	-0.36% (0.29%)	0.184 (0.004)	0.149 (0.0002)
10	Gamma(2,5)	0.002 (0.005)	0.09% (0.24%)	0.151 (0.003)	0.148 (0.0001)
3	Gamma(2,20)	-0.098 (0.005)	-4.97% (0.26%)	0.164 (0.004)	0.140 (0.0002)
6	Gamma(2,20)	-0.001 (0.006)	-0.04% (0.30%)	0.185 (0.004)	0.149 (0.0002)
10	Gamma(2,20)	0.008 (0.005)	0.43% (0.24%)	0.152 (0.003)	0.148 (0.0001)

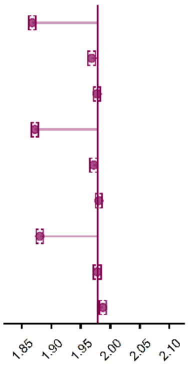

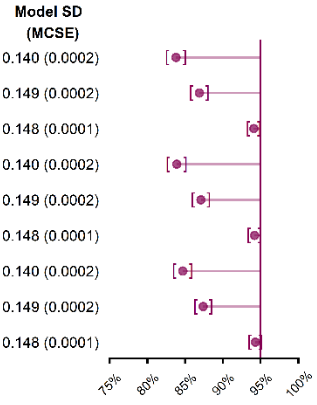

Supplementary Figure 7: Prior and posterior distributions of the smoothness parameter σ across scenarios for cetuximab OS and nivolumab PFS, with an exchangeable prior on the spline coefficients. Posterior is shown for models fitted from 50 simulated datasets.

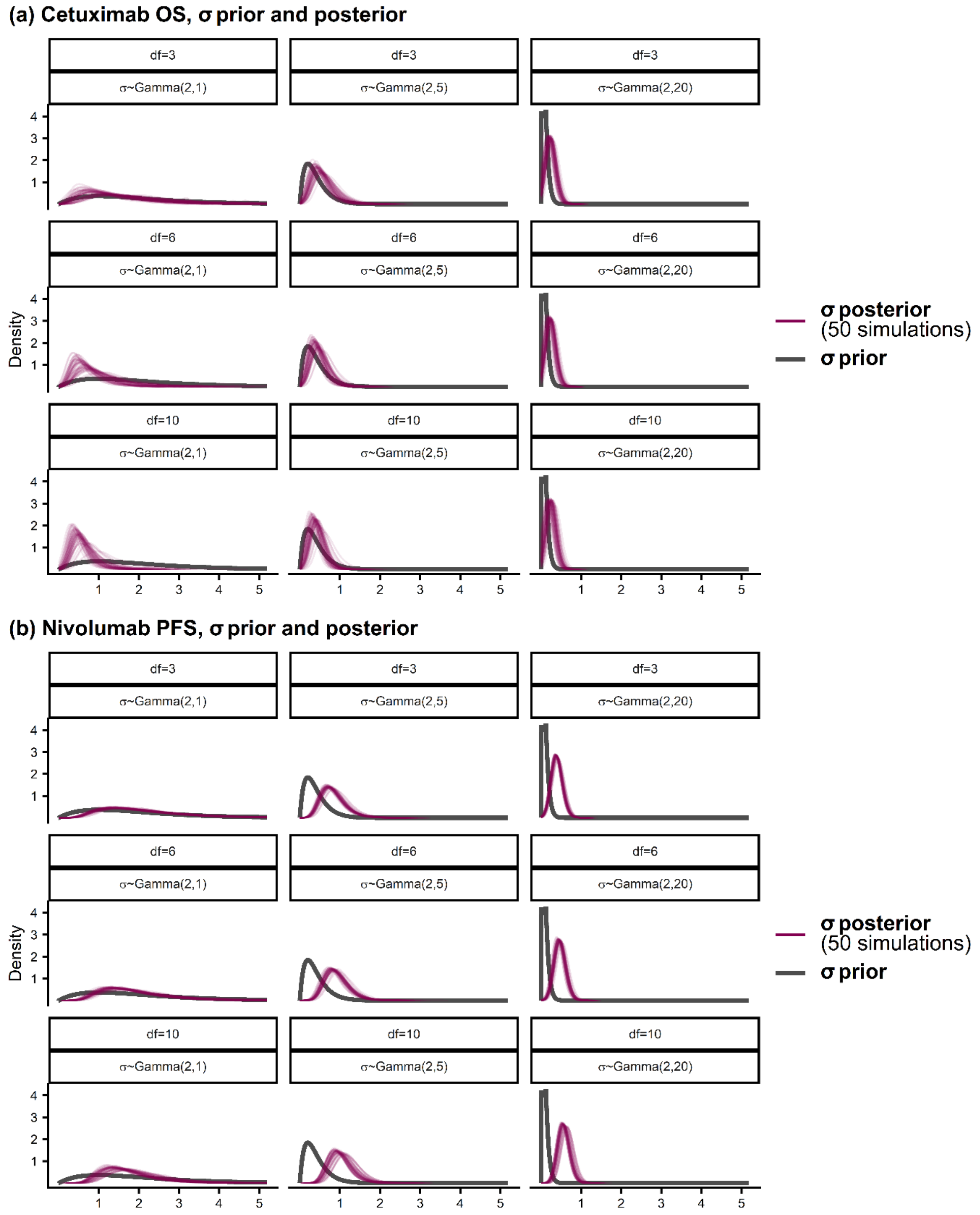

Supplementary Figure 8: Stan warning messages and convergence diagnostics for modelling a single treatment arm using a random walk prior on the spline coefficients.

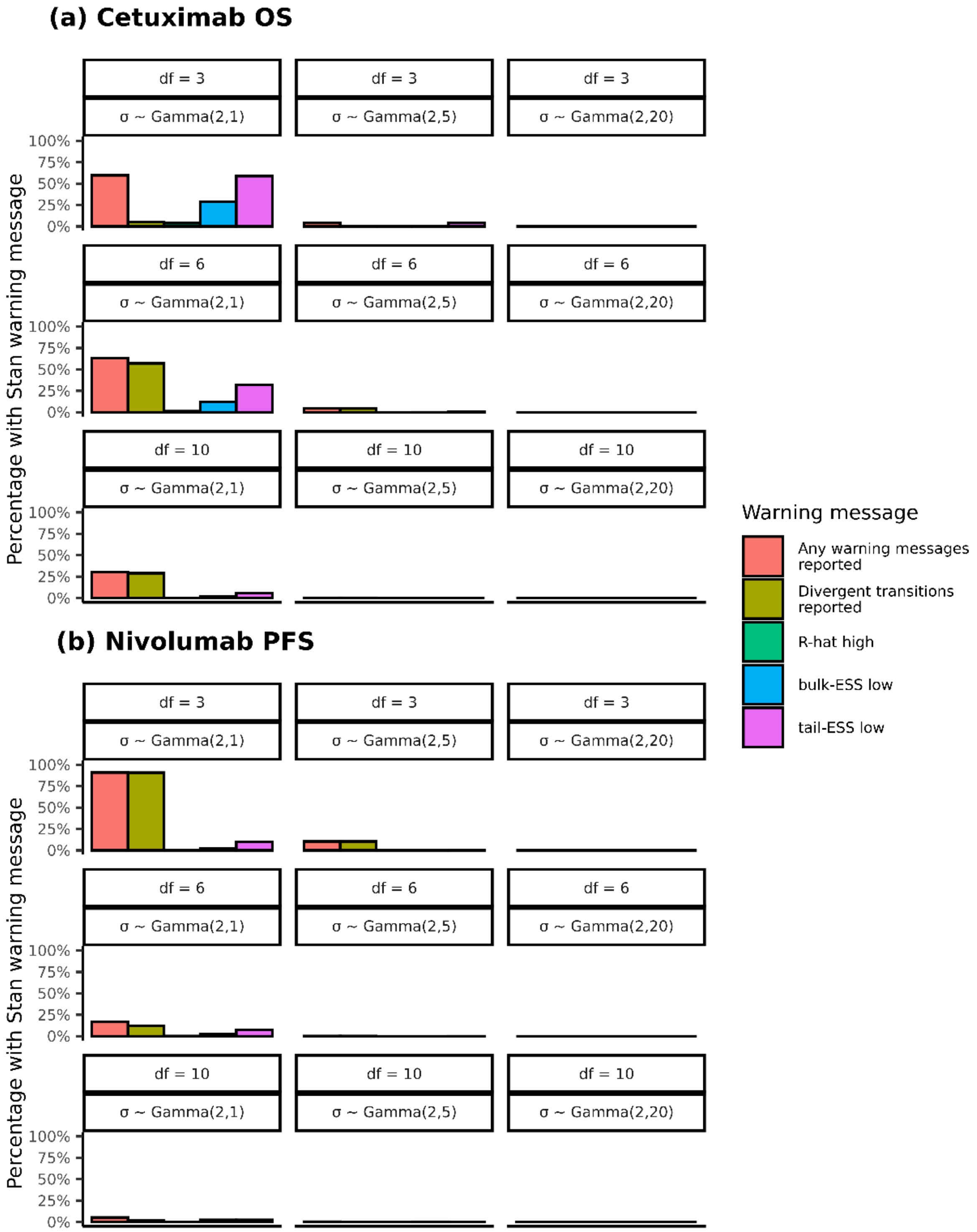

Supplementary Figure 9: Stan warning messages and convergence diagnostics for modelling a single treatment arm using an exchangeable prior on the spline coefficients.

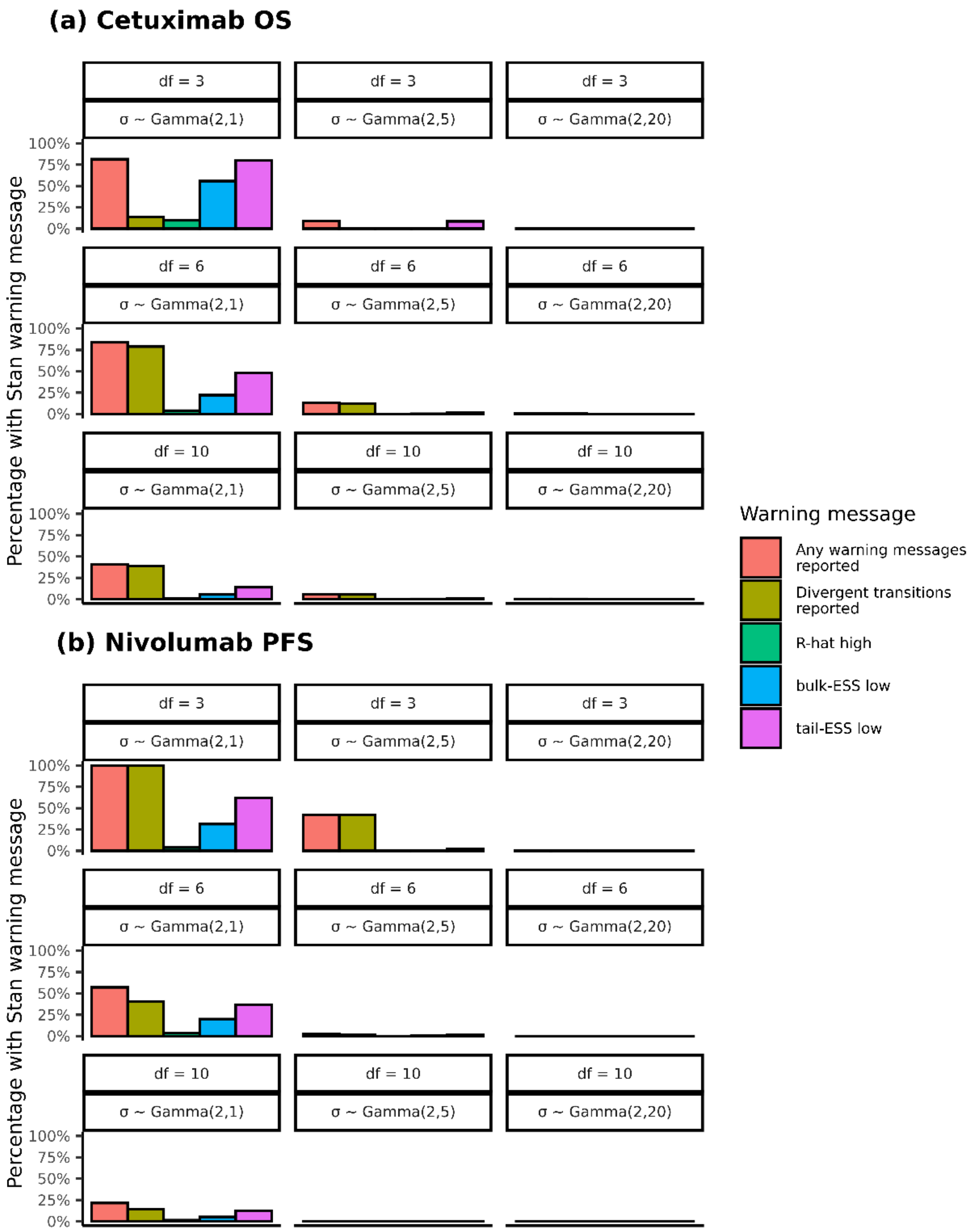

Supplementary Figure 10: Survival and hazard plots for a `survextrap` model fitted using a random walk prior on the spline coefficients based on 50 simulated datasets from the Nivolumab PFS case study.

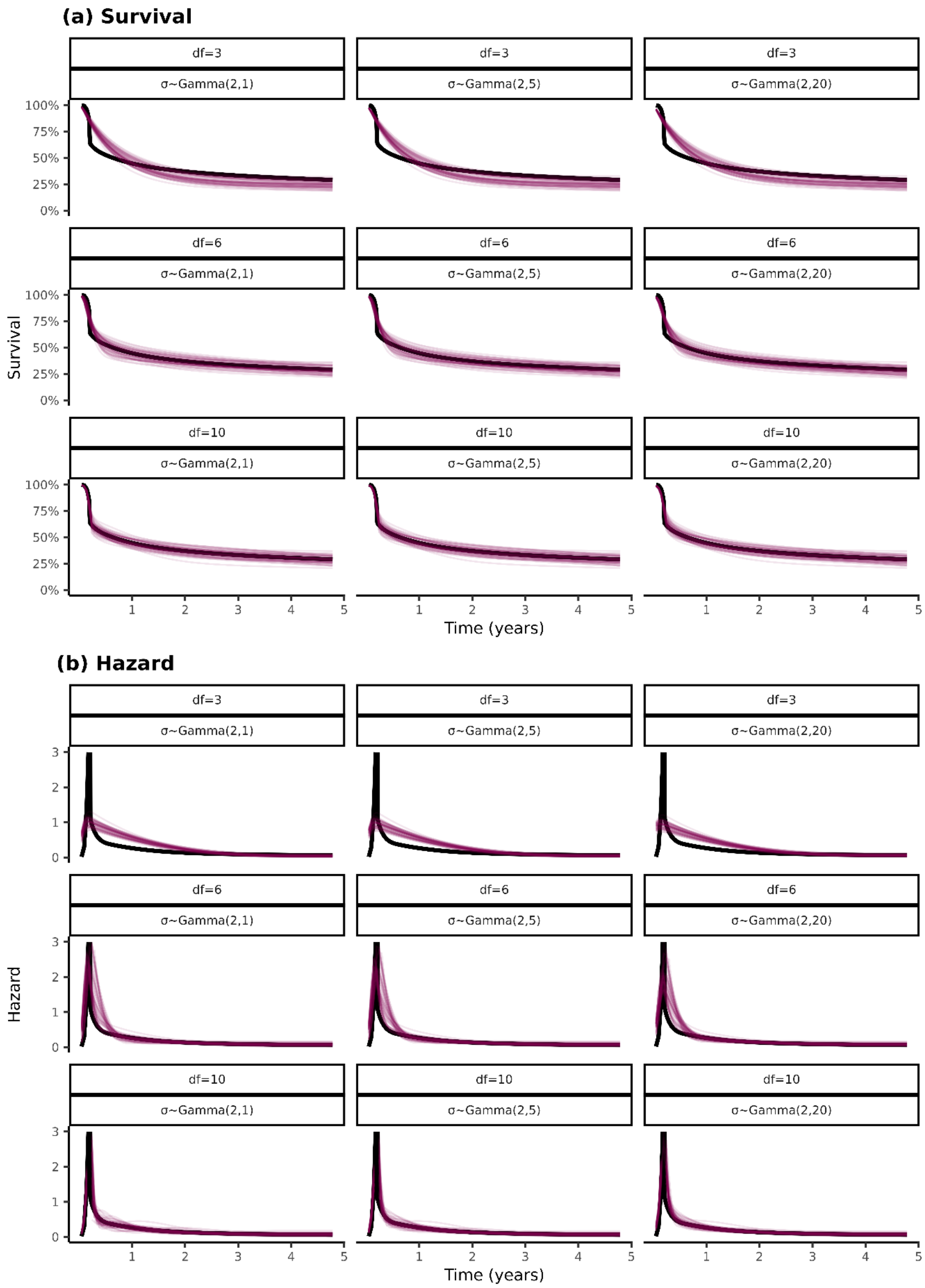

The black line shows the true survival and hazard function, the maroon lines show the model estimates.

Supplementary Figure 11: Survival and hazard plots for a `survextrap` model fitted using an exchangeable prior on the spline coefficients based on 50 simulated datasets from the Nivolumab PFS case study.

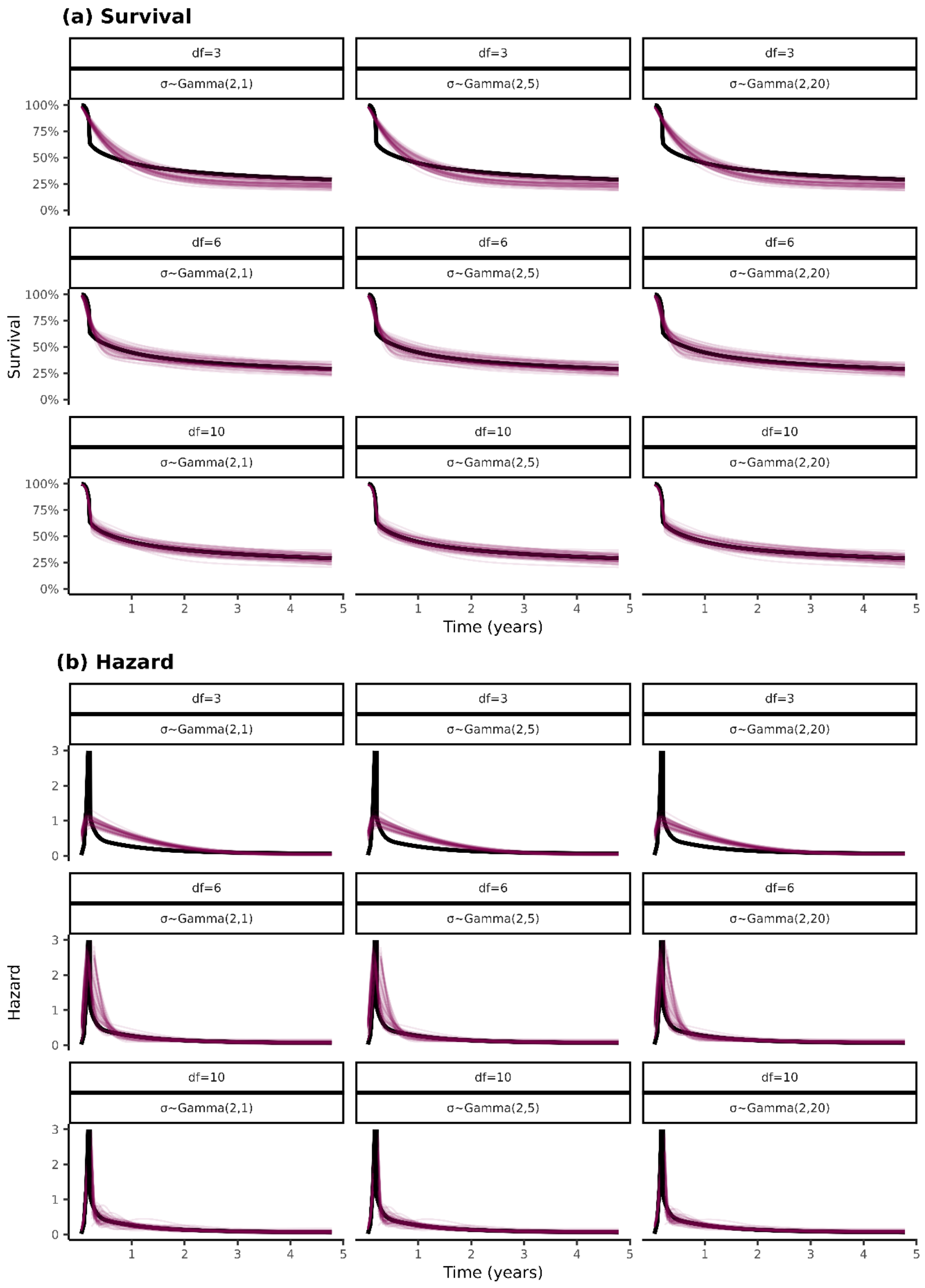

The black line shows the true survival and hazard function, the maroon lines show the model estimates.

Supplementary Figure 12: Performance measures for the posterior median RMST at 5-y based on simulated data from the Cetuximab OS and Nivolumab PFS case studies using a Laplace approximation method (`fit_method = "opt"`) for `survextrap` and a random walk prior.

(a) Cetuximab OS

M-spline df	σ prior	Bias (MCSE)	Relative bias (%) (MCSE)	Empirical SE (MCSE)	Model SD (MCSE)
3	Gamma(2,1)	-0.030 (0.004)	-0.94% (0.14%)	0.136 (0.003)	0.140 (0.0001)
6	Gamma(2,1)	0.010 (0.004)	0.30% (0.14%)	0.137 (0.003)	0.144 (0.0001)
10	Gamma(2,1)	0.016 (0.004)	0.51% (0.13%)	0.135 (0.003)	0.155 (0.0003)
3	Gamma(2,5)	-0.004 (0.004)	-0.12% (0.14%)	0.137 (0.003)	0.135 (0.0001)
6	Gamma(2,5)	0.009 (0.004)	0.28% (0.14%)	0.137 (0.003)	0.137 (0.0001)
10	Gamma(2,5)	0.017 (0.004)	0.52% (0.13%)	0.136 (0.003)	0.141 (0.0002)
3	Gamma(2,20)	0.057 (0.005)	1.78% (0.14%)	0.145 (0.003)	0.136 (0.0001)
6	Gamma(2,20)	0.045 (0.004)	1.42% (0.14%)	0.142 (0.003)	0.136 (0.0001)
10	Gamma(2,20)	0.046 (0.004)	1.45% (0.14%)	0.140 (0.003)	0.139 (0.0003)

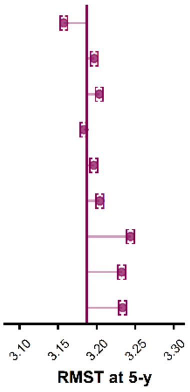

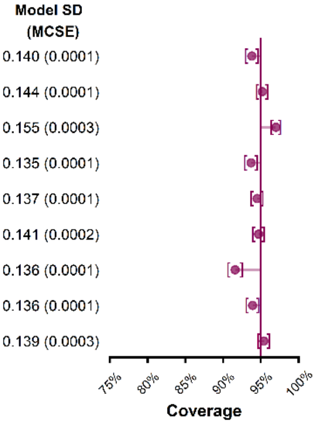

(b) Nivolumab PFS

M-spline df	σ prior	Bias (MCSE)	Relative bias (%) (MCSE)	Empirical SE (MCSE)	Model SD (MCSE)
3	Gamma(2,1)	-0.130 (0.005)	-6.57% (0.26%)	0.161 (0.004)	0.143 (0.0002)
6	Gamma(2,1)	-0.013 (0.006)	-0.66% (0.29%)	0.182 (0.004)	0.150 (0.0002)
10	Gamma(2,1)	-0.001 (0.005)	-0.04% (0.25%)	0.154 (0.003)	0.151 (0.0002)
3	Gamma(2,5)	-0.111 (0.005)	-5.61% (0.26%)	0.162 (0.004)	0.141 (0.0002)
6	Gamma(2,5)	-0.023 (0.006)	-1.16% (0.28%)	0.178 (0.004)	0.149 (0.0002)
10	Gamma(2,5)	-0.004 (0.005)	-0.18% (0.25%)	0.154 (0.003)	0.149 (0.0001)
3	Gamma(2,20)	-0.081 (0.005)	-4.07% (0.26%)	0.162 (0.004)	0.141 (0.0002)
6	Gamma(2,20)	-0.031 (0.005)	-1.59% (0.27%)	0.170 (0.004)	0.148 (0.0001)
10	Gamma(2,20)	-0.002 (0.005)	-0.10% (0.25%)	0.155 (0.003)	0.148 (0.0001)

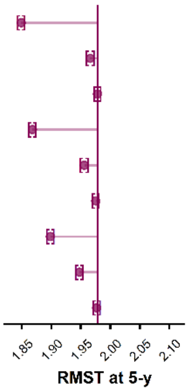

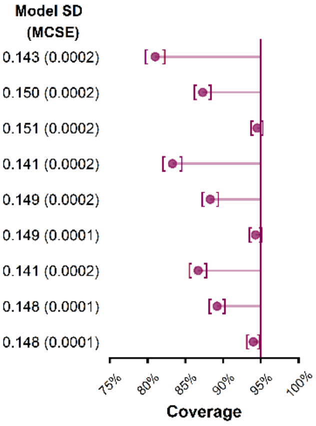

Supplementary Figure 13: Performance measures for the posterior median RMST at 2- and 3-y based on simulated data from the Cetuximab OS and Nivolumab PFS case studies with shorter follow-up of 2 and 3 years, respectively. Models use a random walk prior. Highlighted rows show the default settings in `survextrap`.

(a) Cetuximab OS, 2-year data cut

M-spline df	σ prior	Bias (MCSE)	Relative bias (%) (MCSE)	Empirical SE (MCSE)	Model SD (MCSE)
3	Gamma(2,1)	0.015 (0.001)	0.96% (0.08%)	0.042 (0.001)	0.043 (0.0001)
6	Gamma(2,1)	0.002 (0.001)	0.13% (0.08%)	0.042 (0.001)	0.042 (0.0001)
10	Gamma(2,1)	0.001 (0.001)	0.08% (0.08%)	0.042 (0.001)	0.042 (0.0001)
3	Gamma(2,5)	0.015 (0.001)	0.92% (0.08%)	0.040 (0.001)	0.042 (0.0001)
6	Gamma(2,5)	0.003 (0.001)	0.20% (0.08%)	0.041 (0.001)	0.042 (0.0001)
10	Gamma(2,5)	0.002 (0.001)	0.12% (0.08%)	0.041 (0.001)	0.042 (0.0001)
3	Gamma(2,20)	0.007 (0.001)	0.43% (0.08%)	0.040 (0.001)	0.040 (0.0001)
6	Gamma(2,20)	0.004 (0.001)	0.23% (0.08%)	0.040 (0.001)	0.041 (0.0001)
10	Gamma(2,20)	0.003 (0.001)	0.16% (0.08%)	0.040 (0.001)	0.041 (0.0001)

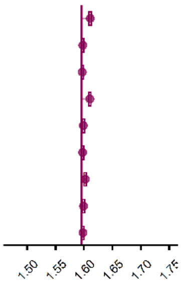

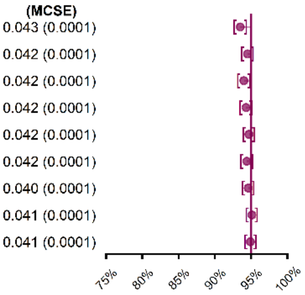

(b) Cetuximab OS, 3-year data cut

M-spline df	σ prior	Bias (MCSE)	Relative bias (%) (MCSE)	Empirical SE (MCSE)	Model SD (MCSE)
3	Gamma(2,1)	-0.021 (0.002)	-0.94% (0.11%)	0.078 (0.002)	0.076 (0.0001)
6	Gamma(2,1)	-0.010 (0.002)	-0.47% (0.11%)	0.077 (0.002)	0.076 (0.0001)
10	Gamma(2,1)	-0.010 (0.002)	-0.45% (0.11%)	0.077 (0.002)	0.076 (0.0001)
3	Gamma(2,5)	-0.023 (0.002)	-1.06% (0.11%)	0.078 (0.002)	0.076 (0.0001)
6	Gamma(2,5)	-0.015 (0.002)	-0.69% (0.11%)	0.077 (0.002)	0.076 (0.0001)
10	Gamma(2,5)	-0.015 (0.002)	-0.67% (0.11%)	0.077 (0.002)	0.076 (0.0001)
3	Gamma(2,20)	-0.027 (0.002)	-1.22% (0.11%)	0.078 (0.002)	0.077 (0.0001)
6	Gamma(2,20)	-0.025 (0.002)	-1.13% (0.11%)	0.078 (0.002)	0.077 (0.0001)
10	Gamma(2,20)	-0.024 (0.002)	-1.11% (0.11%)	0.078 (0.002)	0.077 (0.0001)

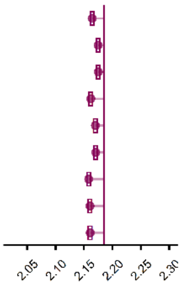

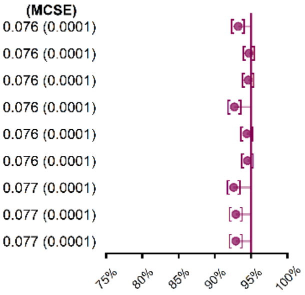

(c) Nivolumab PFS, 2-year data cut

M-spline df	σ prior	Bias (MCSE)	Relative bias (%) (MCSE)	Empirical SE (MCSE)	Model SD (MCSE)
3	Gamma(2,1)	-0.005 (0.002)	-0.47% (0.19%)	0.061 (0.001)	0.058 (0.0000)
6	Gamma(2,1)	-0.013 (0.002)	-1.29% (0.19%)	0.062 (0.001)	0.059 (0.0000)
10	Gamma(2,1)	-0.000 (0.002)	-0.04% (0.19%)	0.061 (0.001)	0.059 (0.0000)
3	Gamma(2,5)	-0.002 (0.002)	-0.19% (0.19%)	0.061 (0.001)	0.058 (0.0000)
6	Gamma(2,5)	-0.013 (0.002)	-1.31% (0.19%)	0.062 (0.001)	0.059 (0.0000)
10	Gamma(2,5)	-0.001 (0.002)	-0.11% (0.19%)	0.061 (0.001)	0.059 (0.0000)
3	Gamma(2,20)	0.006 (0.002)	0.56% (0.19%)	0.061 (0.001)	0.058 (0.0000)
6	Gamma(2,20)	-0.010 (0.002)	-1.01% (0.19%)	0.062 (0.001)	0.059 (0.0000)
10	Gamma(2,20)	0.000 (0.002)	0.03% (0.19%)	0.061 (0.001)	0.059 (0.0000)

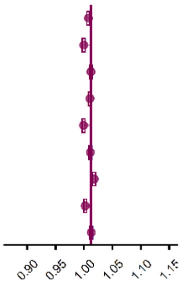

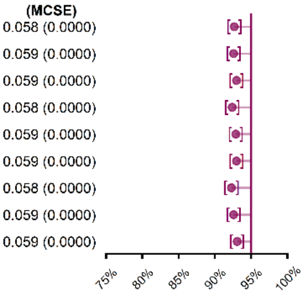

(d) Nivolumab PFS, 3-year data cut

M-spline df	σ prior	Bias (MCSE)	Relative bias (%) (MCSE)	Empirical SE (MCSE)	Model SD (MCSE)
3	Gamma(2,1)	-0.015 (0.003)	-1.09% (0.22%)	0.094 (0.002)	0.090 (0.0001)
6	Gamma(2,1)	-0.039 (0.003)	-2.88% (0.22%)	0.097 (0.002)	0.090 (0.0001)
10	Gamma(2,1)	-0.011 (0.003)	-0.78% (0.22%)	0.094 (0.002)	0.090 (0.0001)
3	Gamma(2,5)	-0.013 (0.003)	-0.99% (0.22%)	0.094 (0.002)	0.090 (0.0001)
6	Gamma(2,5)	-0.037 (0.003)	-2.73% (0.22%)	0.097 (0.002)	0.090 (0.0001)
10	Gamma(2,5)	-0.011 (0.003)	-0.84% (0.22%)	0.094 (0.002)	0.090 (0.0001)
3	Gamma(2,20)	-0.010 (0.003)	-0.73% (0.22%)	0.095 (0.002)	0.089 (0.0001)
6	Gamma(2,20)	-0.033 (0.003)	-2.44% (0.22%)	0.097 (0.002)	0.090 (0.0001)
10	Gamma(2,20)	-0.011 (0.003)	-0.83% (0.22%)	0.094 (0.002)	0.090 (0.0001)

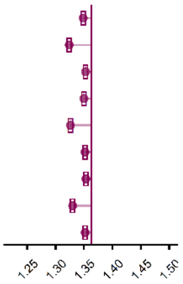

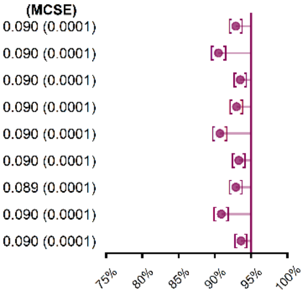

Supplementary Figure 14: Performance measures for the RMST at 5-y based on simulated data from the Cetuximab OS and Nivolumab PFS case studies using frequentist models implemented in R packages `flexsurv` and `rstpm2`.

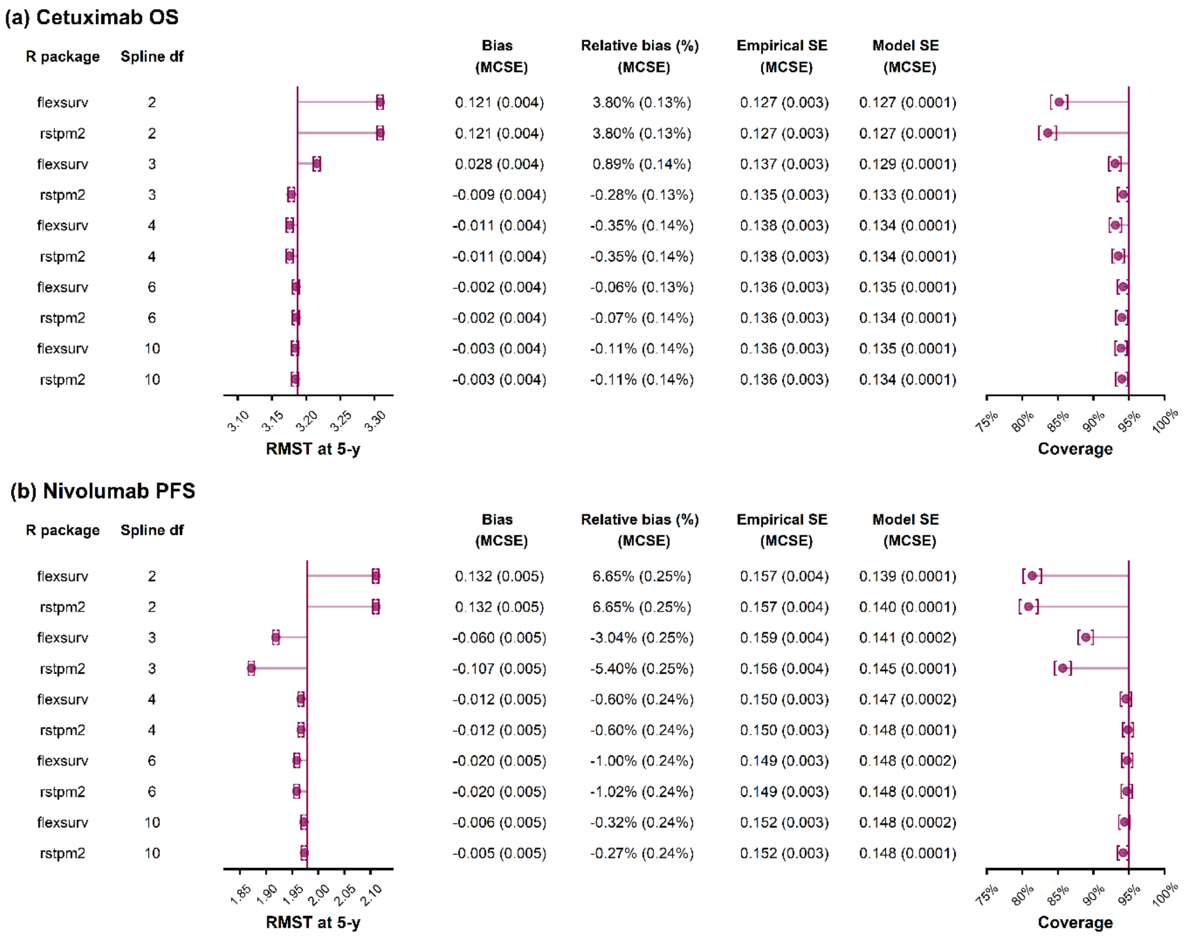

(a) Cetuximab OS

R package	Spline df	Bias (MCSE)	Relative bias (%) (MCSE)	Empirical SE (MCSE)	Model SE (MCSE)
flexsurv	2	0.121 (0.004)	3.80% (0.13%)	0.127 (0.003)	0.127 (0.0001)
rstpm2	2	0.121 (0.004)	3.80% (0.13%)	0.127 (0.003)	0.127 (0.0001)
flexsurv	3	0.028 (0.004)	0.89% (0.14%)	0.137 (0.003)	0.129 (0.0001)
rstpm2	3	-0.009 (0.004)	-0.28% (0.13%)	0.135 (0.003)	0.133 (0.0001)
flexsurv	4	-0.011 (0.004)	-0.35% (0.14%)	0.138 (0.003)	0.134 (0.0001)
rstpm2	4	-0.011 (0.004)	-0.35% (0.14%)	0.138 (0.003)	0.134 (0.0001)
flexsurv	6	-0.002 (0.004)	-0.06% (0.13%)	0.136 (0.003)	0.135 (0.0001)
rstpm2	6	-0.002 (0.004)	-0.07% (0.14%)	0.136 (0.003)	0.134 (0.0001)
flexsurv	10	-0.003 (0.004)	-0.11% (0.14%)	0.136 (0.003)	0.135 (0.0001)
rstpm2	10	-0.003 (0.004)	-0.11% (0.14%)	0.136 (0.003)	0.134 (0.0001)

(b) Nivolumab PFS

R package	Spline df	Bias (MCSE)	Relative bias (%) (MCSE)	Empirical SE (MCSE)	Model SE (MCSE)
flexsurv	2	0.132 (0.005)	6.65% (0.25%)	0.157 (0.004)	0.139 (0.0001)
rstpm2	2	0.132 (0.005)	6.65% (0.25%)	0.157 (0.004)	0.140 (0.0001)
flexsurv	3	-0.060 (0.005)	-3.04% (0.25%)	0.159 (0.004)	0.141 (0.0002)
rstpm2	3	-0.107 (0.005)	-5.40% (0.25%)	0.156 (0.004)	0.145 (0.0001)
flexsurv	4	-0.012 (0.005)	-0.60% (0.24%)	0.150 (0.003)	0.147 (0.0002)
rstpm2	4	-0.012 (0.005)	-0.60% (0.24%)	0.150 (0.003)	0.148 (0.0001)
flexsurv	6	-0.020 (0.005)	-1.00% (0.24%)	0.149 (0.003)	0.148 (0.0002)
rstpm2	6	-0.020 (0.005)	-1.02% (0.24%)	0.149 (0.003)	0.148 (0.0001)
flexsurv	10	-0.006 (0.005)	-0.32% (0.24%)	0.152 (0.003)	0.148 (0.0002)
rstpm2	10	-0.005 (0.005)	-0.27% (0.24%)	0.152 (0.003)	0.148 (0.0001)

Supplementary Figure 15: Performance measures for the difference in RMST at 5-y based on simulated data from the Cetuximab OS case study for the control arm and under four scenarios for the time-varying treatment effect, investigating fitted `survextrap` models that use an exchangeable prior and a default Gamma(2,1) prior for $\boldsymbol{\sigma}$ and where the degrees of freedom and prior for $\boldsymbol{\tau}$ are varied.

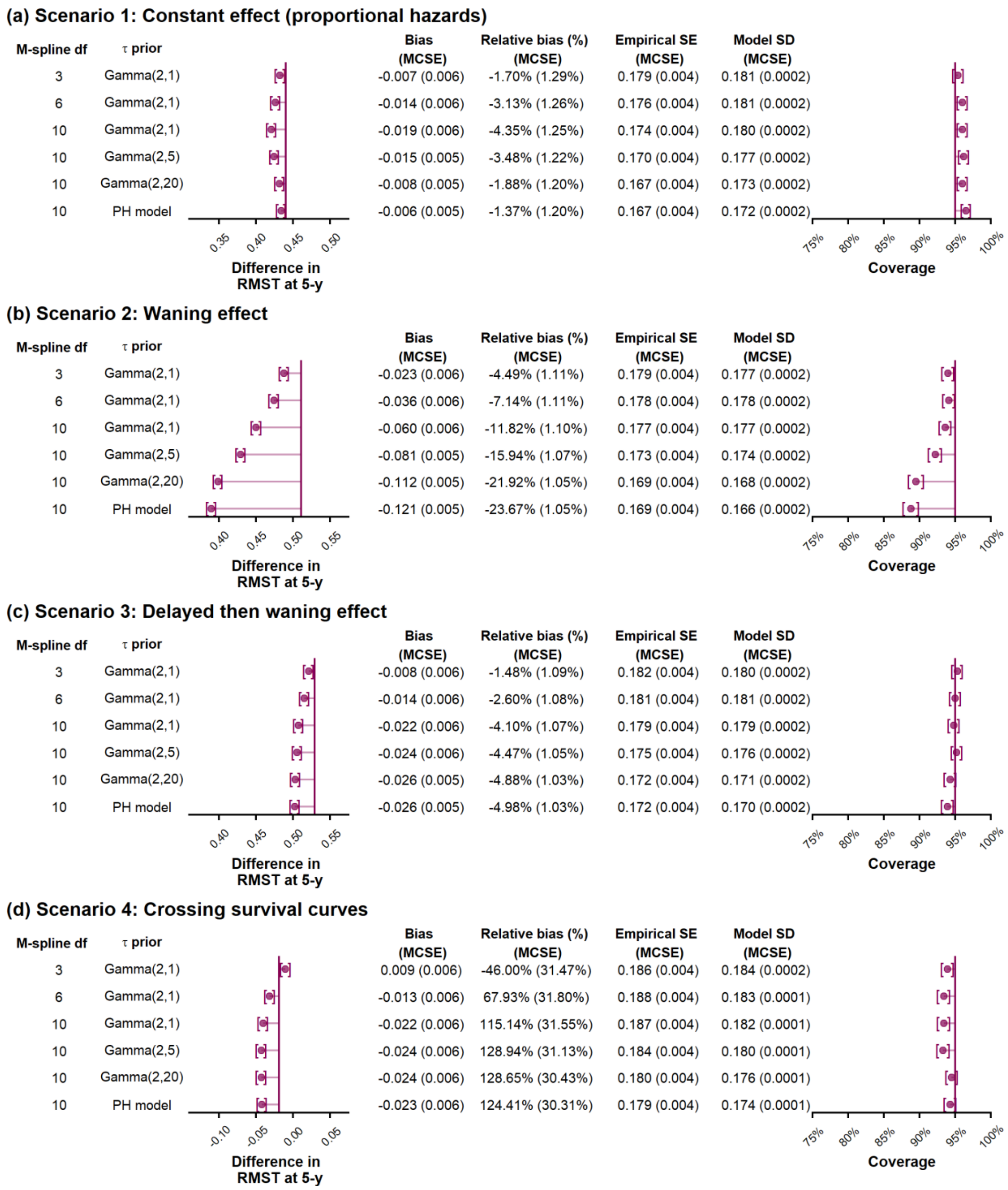

Supplementary Figure 16: Hazard ratio plots for a non-proportional hazards `survextrap` model fitted using an exchangeable prior and varying the degrees of freedom and non-proportionality smoothness prior $\boldsymbol{\tau}$, based on 50 simulated datasets under four treatment-effect scenarios.

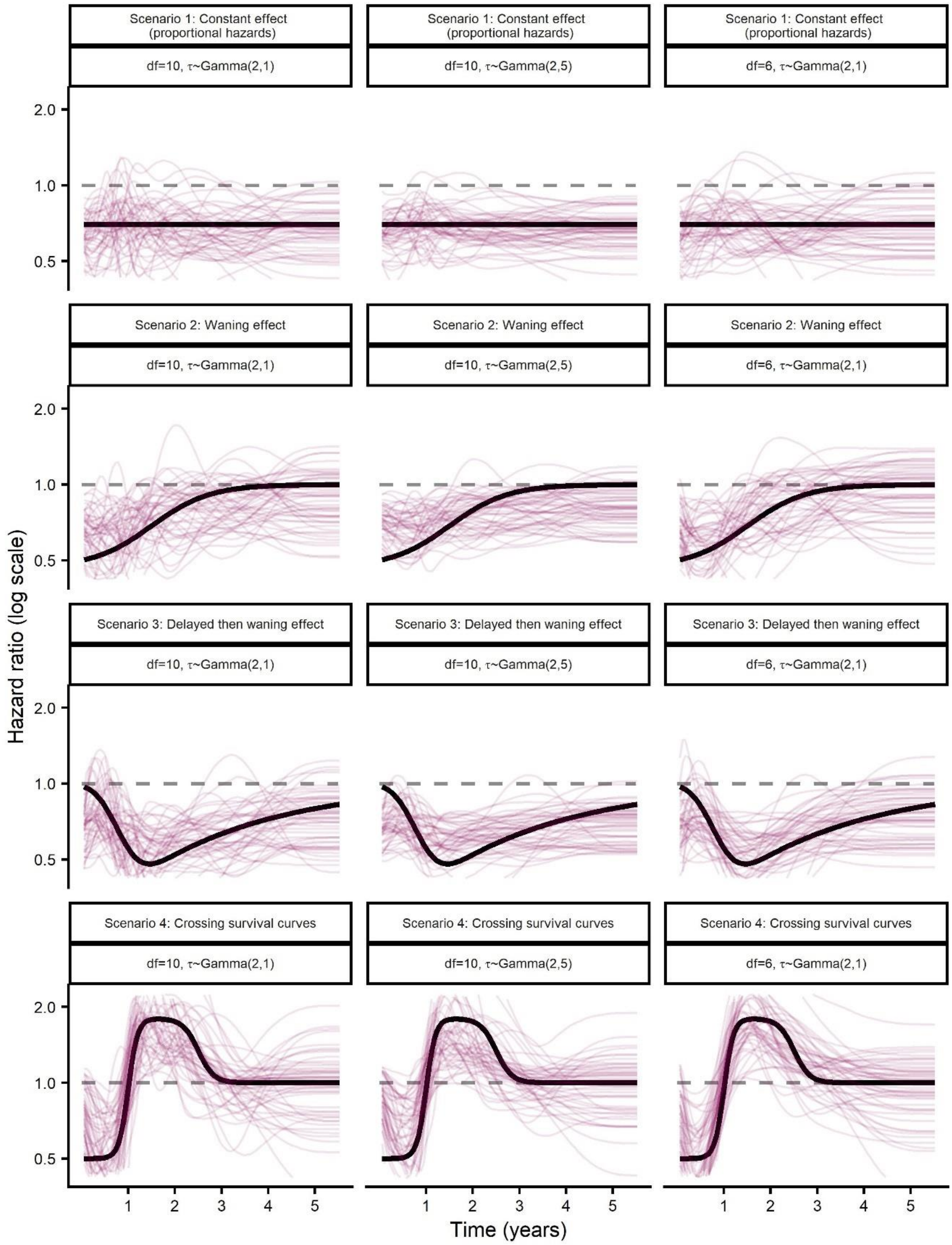

Supplementary Figure 17: Performance measures of frequentist methods for estimating the difference in RMST at 5-y (Scenario 4 overleaf).

(a) Scenario 1: Constant effect (proportional hazards)

R Package	Baseline spline, df	Time-varying effect spline, df	Bias (MCSE)	Relative bias (%) (MCSE)	Empirical SE (MCSE)	Model SE (MCSE)
rstpm2	2	2	-0.022 (0.005)	-4.93% (1.19%)	0.166 (0.004)	0.174 (0.0001)
flexsurv	2	2	-0.022 (0.005)	-4.91% (1.19%)	0.166 (0.004)	0.174 (0.0002)
rstpm2	3	2	-0.003 (0.006)	-0.69% (1.28%)	0.178 (0.004)	0.182 (0.0001)
rstpm2	3	3	-0.005 (0.006)	-1.09% (1.31%)	0.182 (0.004)	0.184 (0.0001)
flexsurv	3	3	-0.005 (0.006)	-1.17% (1.30%)	0.180 (0.004)	0.178 (0.0002)
rstpm2	4	2	-0.002 (0.006)	-0.57% (1.29%)	0.179 (0.004)	0.182 (0.0001)
rstpm2	4	3	-0.005 (0.006)	-1.09% (1.34%)	0.186 (0.004)	0.184 (0.0001)
rstpm2	4	4	-0.004 (0.006)	-0.88% (1.32%)	0.184 (0.004)	0.184 (0.0001)
flexsurv	4	4	-0.004 (0.006)	-0.88% (1.32%)	0.184 (0.004)	0.185 (0.0002)
rstpm2	6	2	-0.003 (0.006)	-0.71% (1.28%)	0.178 (0.004)	0.181 (0.0001)
rstpm2	6	3	-0.005 (0.006)	-1.24% (1.30%)	0.181 (0.004)	0.183 (0.0001)
rstpm2	6	4	-0.006 (0.006)	-1.37% (1.32%)	0.184 (0.004)	0.185 (0.0001)
rstpm2	6	6	-0.006 (0.006)	-1.29% (1.32%)	0.184 (0.004)	0.185 (0.0001)
flexsurv	6	6	-0.006 (0.006)	-1.29% (1.32%)	0.184 (0.004)	0.186 (0.0002)
rstpm2	10	2	-0.003 (0.006)	-0.71% (1.28%)	0.178 (0.004)	0.181 (0.0001)
rstpm2	10	3	-0.005 (0.006)	-1.21% (1.30%)	0.181 (0.004)	0.183 (0.0001)
rstpm2	10	4	-0.006 (0.006)	-1.45% (1.32%)	0.184 (0.004)	0.185 (0.0001)
rstpm2	10	6	-0.005 (0.006)	-1.24% (1.32%)	0.184 (0.004)	0.185 (0.0001)
rstpm2	10	10	-0.005 (0.006)	-1.20% (1.33%)	0.184 (0.004)	0.186 (0.0001)
flexsurv	10	10	-0.005 (0.006)	-1.19% (1.33%)	0.184 (0.004)	0.184 (0.0003)

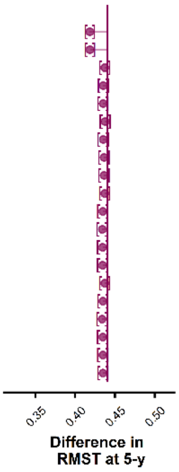

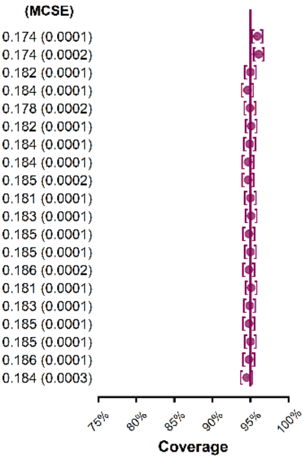

(b) Scenario 2: Waning effect

R Package	Baseline spline, df	Time-varying effect spline, df	Bias (MCSE)	Relative bias (%) (MCSE)	Empirical SE (MCSE)	Model SE (MCSE)
rstpm2	2	2	-0.063 (0.005)	-12.36% (1.04%)	0.168 (0.004)	0.171 (0.0001)
flexsurv	2	2	-0.063 (0.005)	-12.36% (1.04%)	0.168 (0.004)	0.171 (0.0002)
rstpm2	3	2	-0.015 (0.006)	-2.93% (1.11%)	0.179 (0.004)	0.177 (0.0001)
rstpm2	3	3	-0.006 (0.006)	-1.16% (1.12%)	0.181 (0.004)	0.179 (0.0001)
flexsurv	3	3	-0.029 (0.006)	-5.73% (1.11%)	0.179 (0.004)	0.174 (0.0002)
rstpm2	4	2	-0.016 (0.006)	-3.21% (1.11%)	0.179 (0.004)	0.177 (0.0001)
rstpm2	4	3	-0.009 (0.006)	-1.69% (1.12%)	0.180 (0.004)	0.179 (0.0001)
rstpm2	4	4	-0.005 (0.006)	-1.04% (1.13%)	0.182 (0.004)	0.180 (0.0001)
flexsurv	4	4	-0.005 (0.006)	-1.04% (1.13%)	0.182 (0.004)	0.180 (0.0002)
rstpm2	6	2	-0.017 (0.006)	-3.26% (1.11%)	0.180 (0.004)	0.177 (0.0001)
rstpm2	6	3	-0.008 (0.006)	-1.53% (1.11%)	0.180 (0.004)	0.179 (0.0001)
rstpm2	6	4	-0.006 (0.006)	-1.15% (1.13%)	0.182 (0.004)	0.181 (0.0001)
rstpm2	6	6	-0.008 (0.006)	-1.54% (1.13%)	0.182 (0.004)	0.181 (0.0001)
flexsurv	6	6	-0.008 (0.006)	-1.51% (1.13%)	0.182 (0.004)	0.181 (0.0002)
rstpm2	10	2	-0.017 (0.006)	-3.25% (1.11%)	0.180 (0.004)	0.177 (0.0001)
rstpm2	10	3	-0.008 (0.006)	-1.48% (1.12%)	0.180 (0.004)	0.179 (0.0001)
rstpm2	10	4	-0.006 (0.006)	-1.16% (1.13%)	0.182 (0.004)	0.181 (0.0001)
rstpm2	10	6	-0.008 (0.006)	-1.48% (1.13%)	0.182 (0.004)	0.181 (0.0001)
rstpm2	10	10	-0.008 (0.006)	-1.56% (1.13%)	0.182 (0.004)	0.181 (0.0001)
flexsurv	10	10	-0.008 (0.006)	-1.57% (1.13%)	0.182 (0.004)	0.179 (0.0004)

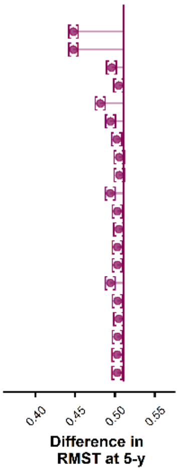

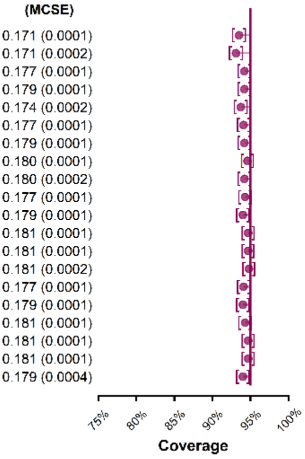

(c) Scenario 3: Delayed then waning effect

R Package	Baseline spline, df	Time-varying effect spline, df	Bias (MCSE)	Relative bias (%) (MCSE)	Empirical SE (MCSE)	Model SE (MCSE)
rstpm2	2	2	-0.056 (0.005)	-10.59% (1.03%)	0.172 (0.004)	0.173 (0.0001)
flexsurv	2	2	-0.056 (0.005)	-10.59% (1.03%)	0.172 (0.004)	0.174 (0.0002)
rstpm2	3	2	-0.041 (0.006)	-7.78% (1.10%)	0.185 (0.004)	0.181 (0.0001)
rstpm2	3	3	-0.015 (0.006)	-2.74% (1.11%)	0.186 (0.004)	0.183 (0.0001)
flexsurv	3	3	-0.009 (0.006)	-1.79% (1.10%)	0.183 (0.004)	0.178 (0.0002)
rstpm2	4	2	-0.045 (0.006)	-8.53% (1.11%)	0.185 (0.004)	0.181 (0.0001)
rstpm2	4	3	-0.017 (0.006)	-3.25% (1.11%)	0.186 (0.004)	0.183 (0.0001)
rstpm2	4	4	-0.001 (0.006)	-0.10% (1.12%)	0.187 (0.004)	0.184 (0.0001)
flexsurv	4	4	-0.001 (0.006)	-0.10% (1.12%)	0.187 (0.004)	0.184 (0.0002)
rstpm2	6	2	-0.052 (0.006)	-9.86% (1.11%)	0.185 (0.004)	0.181 (0.0001)
rstpm2	6	3	-0.027 (0.006)	-5.11% (1.11%)	0.185 (0.004)	0.183 (0.0001)
rstpm2	6	4	-0.000 (0.006)	-0.07% (1.12%)	0.187 (0.004)	0.185 (0.0001)
rstpm2	6	6	-0.001 (0.006)	-0.16% (1.11%)	0.186 (0.004)	0.185 (0.0001)
flexsurv	6	6	-0.001 (0.006)	-0.16% (1.11%)	0.186 (0.004)	0.185 (0.0002)
rstpm2	10	2	-0.052 (0.006)	-9.83% (1.11%)	0.186 (0.004)	0.181 (0.0001)
rstpm2	10	3	-0.027 (0.006)	-5.05% (1.11%)	0.185 (0.004)	0.183 (0.0001)
rstpm2	10	4	-0.001 (0.006)	-0.15% (1.12%)	0.187 (0.004)	0.185 (0.0001)
rstpm2	10	6	0.000 (0.006)	0.07% (1.15%)	0.192 (0.004)	0.185 (0.0001)
rstpm2	10	10	0.000 (0.006)	0.01% (1.11%)	0.186 (0.004)	0.185 (0.0001)
flexsurv	10	10	0.000 (0.006)	0.08% (1.12%)	0.187 (0.004)	0.184 (0.0003)

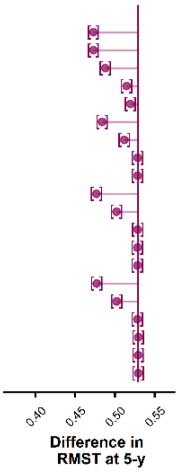

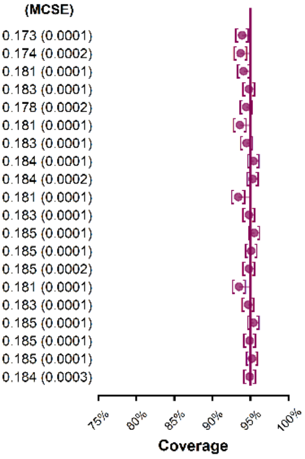

(d) Scenario 4: Crossing survival curves

R Package	Baseline spline, df	Time-varying effect spline, df	Bias (MCSE)	Relative bias (%) (MCSE)	Empirical SE (MCSE)	Model SE (MCSE)
rstpm2	2	2	0.032 (0.006)	-168.99% (29.92%)	0.177 (0.004)	0.176 (0.0001)
flexsurv	2	2	0.032 (0.006)	-168.60% (29.95%)	0.177 (0.004)	0.176 (0.0001)
rstpm2	3	2	0.067 (0.006)	-356.95% (31.46%)	0.186 (0.004)	0.182 (0.0001)
rstpm2	3	3	0.018 (0.006)	-96.03% (31.91%)	0.189 (0.004)	0.183 (0.0001)
flexsurv	3	3	0.018 (0.006)	-95.93% (31.91%)	0.189 (0.004)	0.183 (0.0002)
rstpm2	4	2	0.053 (0.006)	-285.76% (31.84%)	0.188 (0.004)	0.182 (0.0001)
rstpm2	4	3	0.016 (0.006)	-86.45% (32.07%)	0.190 (0.004)	0.183 (0.0001)
rstpm2	4	4	-0.005 (0.006)	25.47% (32.29%)	0.191 (0.004)	0.183 (0.0001)
flexsurv	4	4	-0.005 (0.006)	25.46% (32.29%)	0.191 (0.004)	0.183 (0.0002)
rstpm2	6	2	0.068 (0.006)	-366.02% (31.72%)	0.188 (0.004)	0.182 (0.0001)
rstpm2	6	3	0.030 (0.006)	-162.73% (31.84%)	0.188 (0.004)	0.183 (0.0001)
rstpm2	6	4	-0.010 (0.006)	55.59% (32.41%)	0.192 (0.004)	0.184 (0.0001)
rstpm2	6	6	-0.002 (0.006)	8.16% (32.89%)	0.195 (0.004)	0.185 (0.0001)
flexsurv	6	6	-0.000 (0.006)	1.54% (32.23%)	0.191 (0.004)	0.184 (0.0002)
rstpm2	10	2	0.068 (0.006)	-365.25% (31.70%)	0.187 (0.004)	0.182 (0.0001)
rstpm2	10	3	0.031 (0.006)	-164.65% (31.88%)	0.188 (0.004)	0.183 (0.0001)
rstpm2	10	4	-0.010 (0.006)	52.57% (32.29%)	0.191 (0.004)	0.184 (0.0001)
rstpm2	10	6	-0.000 (0.006)	1.06% (32.25%)	0.191 (0.004)	0.185 (0.0001)
rstpm2	10	10	0.001 (0.006)	-3.96% (32.30%)	0.191 (0.004)	0.185 (0.0001)
flexsurv	10	10	0.001 (0.006)	-3.97% (32.28%)	0.191 (0.004)	0.182 (0.0004)

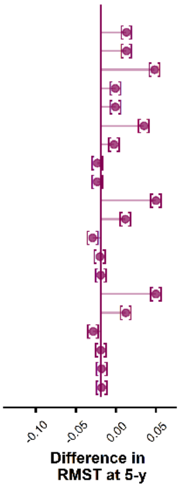

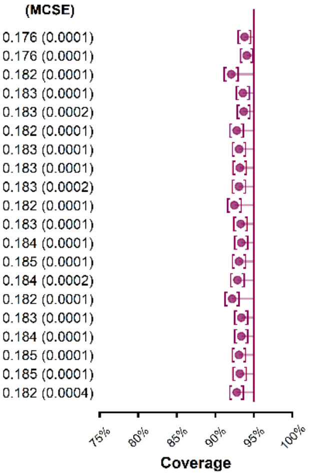

Supplementary Figure 18: Performance measures for estimating 5-y RMST using the standard M-spline basis functions (unsmoothed at the upper boundary knot, option `bsmooth = FALSE`) and smoothed basis functions (smoothed at upper boundary knot, option `bsmooth = TRUE`). Models fitted using a random walk prior.

(a) Cetuximab OS

Basis	M-spline df	σ prior	Bias (MCSE)	Relative bias (%) (MCSE)	Empirical SE (MCSE)	Model SD (MCSE)
Smoothed	6	Gamma(2,1)	0.008 (0.004)	0.26% (0.14%)	0.136 (0.003)	0.134 (0.0001)
Standard	6	Gamma(2,1)	0.008 (0.004)	0.24% (0.14%)	0.138 (0.003)	0.133 (0.0001)
Smoothed	6	Gamma(2,5)	0.016 (0.004)	0.49% (0.14%)	0.138 (0.003)	0.133 (0.0001)
Standard	6	Gamma(2,5)	0.021 (0.004)	0.66% (0.14%)	0.139 (0.003)	0.133 (0.0001)
Smoothed	6	Gamma(2,20)	0.054 (0.005)	1.71% (0.14%)	0.143 (0.003)	0.133 (0.0001)
Standard	6	Gamma(2,20)	0.070 (0.005)	2.21% (0.14%)	0.144 (0.003)	0.133 (0.0002)
Smoothed	10	Gamma(2,1)	0.007 (0.004)	0.23% (0.14%)	0.136 (0.003)	0.134 (0.0001)
Standard	10	Gamma(2,1)	0.006 (0.004)	0.19% (0.14%)	0.136 (0.003)	0.134 (0.0001)
Smoothed	10	Gamma(2,5)	0.016 (0.004)	0.49% (0.14%)	0.137 (0.003)	0.134 (0.0001)
Standard	10	Gamma(2,5)	0.016 (0.004)	0.50% (0.14%)	0.138 (0.003)	0.134 (0.0001)
Smoothed	10	Gamma(2,20)	0.054 (0.005)	1.69% (0.14%)	0.142 (0.003)	0.133 (0.0001)
Standard	10	Gamma(2,20)	0.061 (0.005)	1.91% (0.14%)	0.143 (0.003)	0.133 (0.0001)

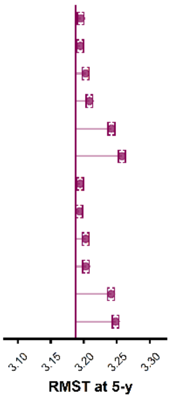

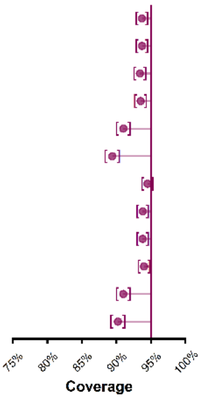

(b) Nivolumab PFS

Basis	M-spline df	σ prior	Bias (MCSE)	Relative bias (%) (MCSE)	Empirical SE (MCSE)	Model SD (MCSE)
Smoothed	6	Gamma(2,1)	-0.018 (0.006)	-0.89% (0.29%)	0.180 (0.004)	0.149 (0.0002)
Standard	6	Gamma(2,1)	0.004 (0.006)	0.22% (0.29%)	0.182 (0.004)	0.149 (0.0002)
Smoothed	6	Gamma(2,5)	-0.023 (0.006)	-1.16% (0.28%)	0.177 (0.004)	0.149 (0.0001)
Standard	6	Gamma(2,5)	-0.014 (0.006)	-0.69% (0.29%)	0.183 (0.004)	0.147 (0.0002)
Smoothed	6	Gamma(2,20)	-0.027 (0.005)	-1.39% (0.27%)	0.169 (0.004)	0.148 (0.0001)
Standard	6	Gamma(2,20)	-0.046 (0.006)	-2.31% (0.28%)	0.175 (0.004)	0.144 (0.0002)
Smoothed	10	Gamma(2,1)	-0.006 (0.005)	-0.29% (0.24%)	0.152 (0.003)	0.148 (0.0001)
Standard	10	Gamma(2,1)	0.016 (0.005)	0.80% (0.25%)	0.157 (0.004)	0.149 (0.0001)
Smoothed	10	Gamma(2,5)	-0.006 (0.005)	-0.28% (0.24%)	0.153 (0.003)	0.149 (0.0001)
Standard	10	Gamma(2,5)	0.009 (0.005)	0.44% (0.25%)	0.156 (0.003)	0.149 (0.0001)
Smoothed	10	Gamma(2,20)	-0.002 (0.005)	-0.09% (0.25%)	0.154 (0.003)	0.148 (0.0001)
Standard	10	Gamma(2,20)	-0.002 (0.005)	-0.09% (0.25%)	0.155 (0.003)	0.148 (0.0001)

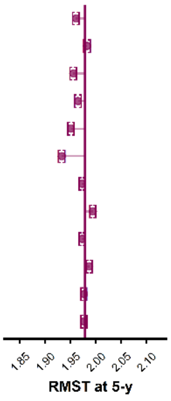

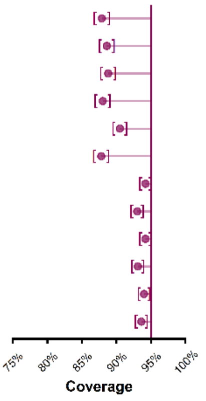

References

[1] D.M. Phillippo, S. Dias, A.E. Ades, N.J. Welton, Multilevel network meta-regression for general likelihoods: synthesis of individual and aggregate data with applications to survival analysis., arXiv, (2024) arXiv.2401.12640.
[2] T.P. Morris, I.R. White, M.J. Crowther, Using simulation studies to evaluate statistical methods, Stat Med, 38 (2019) 2074–2102.
[3] A. Gasparini, rsimsum: Summarise results from Monte Carlo simulation studies, Journal of Open Source Software, 3 (2018) 739.
[4] J.O. Ramsay, Monotone Regression Splines in Action, Statistical Science, 3 (1988) 425–441.
[5] C.H. Jackson, survextrap: a package for flexible and transparent survival extrapolation, BMC Med Res Methodol, 23 (2023) 282.
